# A Rab5 GTPase module is important for autophagosome closure

**DOI:** 10.1371/journal.pgen.1007020

**Published:** 2017-09-21

**Authors:** Fan Zhou, Shenshen Zou, Yong Chen, Zhanna Lipatova, Dan Sun, Xiaolong Zhu, Rui Li, Zulin Wu, Weiming You, Xiaoxia Cong, Yiting Zhou, Zhiping Xie, Valeriya Gyurkovska, Yutao Liu, Qunli Li, Wenjing Li, Jie Cheng, Yongheng Liang, Nava Segev

**Affiliations:** 1 College of Life Sciences, Key Laboratory of Agricultural Environmental Microbiology of Ministry of Agriculture, Nanjing Agricultural University, Nanjing, China; 2 Department of Biochemistry and Molecular Genetics, College of Medicine, University of Illinois at Chicago; 3 Department of Biochemistry and Molecular Biology, Dr. Li Dak Sam & Yap Yio Chin Center for Stem Cell and Regenerative Medicine, Zhejiang University School of Medicine, Hangzhou, China; 4 School of Life Sciences and Technology, Shanghai Jiao Tong University, Shanghai, China; 5 College of Engineering, Nanjing Agricultural University, Nanjing, China; University of Washington, UNITED STATES

## Abstract

In the conserved autophagy pathway, the double-membrane autophagosome (AP) engulfs cellular components to be delivered for degradation in the lysosome. While only sealed AP can productively fuse with the lysosome, the molecular mechanism of AP closure is currently unknown. Rab GTPases, which regulate all intracellular trafficking pathways in eukaryotes, also regulate autophagy. Rabs function in GTPase modules together with their activators and downstream effectors. In yeast, an autophagy-specific Ypt1 GTPase module, together with a set of autophagy-related proteins (Atgs) and a phosphatidylinositol-3-phosphate (PI3P) kinase, regulates AP formation. Fusion of APs and endosomes with the vacuole (the yeast lysosome) requires the Ypt7 GTPase module. We have previously shown that the Rab5-related Vps21, within its endocytic GTPase module, regulates autophagy. However, it was not clear which autophagy step it regulates. Here, we show that this module, which includes the Vps9 activator, the Rab5-related Vps21, the CORVET tethering complex, and the Pep12 SNARE, functions after AP expansion and before AP closure. Whereas APs are not formed in mutant cells depleted for Atgs, sealed APs accumulate in cells depleted for the Ypt7 GTPase module members. Importantly, depletion of individual members of the Vps21 module results in a novel phenotype: accumulation of unsealed APs. In addition, we show that Vps21-regulated AP closure precedes another AP maturation step, the previously reported PI3P phosphatase-dependent Atg dissociation. Our results delineate three successive steps in the autophagy pathway regulated by Rabs, Ypt1, Vps21 and Ypt7, and provide the first insight into the upstream regulation of AP closure.

## Author summary

In autophagy, a cellular recycling pathway, the double-membrane autophagosome (AP) engulfs excess or damaged cargo and delivers it for degradation in the lysosome for the reuse of its building blocks. While plenty of information is currently available regarding AP formation, expansion and fusion, not much is known about the regulation of AP closure, which is required for fusion of APs with the lysosome. Here, we use yeast genetics to characterize a novel mutant phenotype, accumulation of unsealed APs, and identify a role for the Rab5-related Vps21 GTPase in this process. Rab GTPases function in modules that include upstream activators and downstream effectors. We have previously shown that the same Vps21 module that regulates endocytosis also plays a role in autophagy. Using single and double mutant analyses, we find that this module is important for AP closure. Moreover, we delineate three Rab GTPase-regulated steps in the autophagy pathway: AP formation, closure, and fusion, which are regulated by Ypt1, Vps21 and Ypt7, respectively. This study provides the first insight into the mechanism of the elusive process of AP closure.

## Introduction

In autophagy, parts of the cytoplasm, including organelles, are engulfed by the phagophore (or isolation) membrane, which expands and closes to form the double-membrane autophagosomes (APs). APs then fuse with the cellular degradative compartment termed lysosome, and the degradation products are recycled back to the cytoplasm. This process is important for the ability of cells to respond to stress, and is involved in the etiology of multiple human diseases [1, 2]. A set of core autophagy-related proteins, Atgs, were identified in yeast and shown to be conserved from yeast to human cells [3]. These Atgs, together with membrane, assemble to form the pre-autophagosomal structure (PAS), which is required for the formation of the isolation membrane and its expansion. However, currently very little is known about mechanisms that specifically regulate closure of APs, which is required for their successful fusion with the lysosome and the resulting delivery of single-membrane surrounded cargo for degradation [4, 5].

A family of conserved GTPases, eleven Ypts (includes Vps21 and Sec4) in yeast and seventy Rabs in mammalian cells, regulates all membrane-associated intracellular trafficking pathways. These GTPases are activated by their cognate guanine-nucleotide exchange factors, GEFs, and when on membranes in the GTP-bound state, they recruit their downstream effectors. Rab effectors include all the known membrane trafficking machinery components, such as vesicle coats, cytoskeletal motors, tethering factors and SNAREs [6]. Recently, Rab GTPases also emerged as regulators of autophagy [7].

In yeast, Ypt1 is required for ER-to-Golgi transport in the secretory pathway, and is also essential for PAS formation. Interestingly, Ypt1 regulates these two very different processes in the context of two distinct modules, namely, using different GEFs and effectors [8]. Ypt7, which is required in the endocytic pathway for endosome fusion with the vacuole (the yeast lysosome) [9, 10], is also essential in autophagy for fusion of APs with the vacuole. Ypt7, unlike Ypt1, functions in endocytosis and autophagy with the same module of GEF and effectors [11–13]. The yeast proteome contains three Rab5-related proteins: Vps21 (Ypt51), Ypt52 and Ypt53 [14]. A Vps21 GTPase module regulates a step in the endocytic pathway that precedes the one controlled by the Ypt7 GTPase module [9]. A role for Rab5 in autophagy in yeast was originally suggested based on selective and general autophagy defects of *vps21Δ ypt52Δ* double deletion mutant cells (but not of *vps21Δ* single deletion mutant cells) [15]. More recently, based on selective and general autophagy defects of *vps21Δ* (single) deletion cells, we have shown that Vps21 regulates autophagy in the context of its endocytic GTPase module. Specifically, depletion of Vps21, its GEF, or its effectors, results in accumulation of APs [16]. The human Rab1, Rab5 and Rab7 GTPases, homologs of the yeast Ypt1, Vps21 and Ypt7, respectively, were also implicated in autophagy [17].

In addition to membrane sealing, AP maturation also includes the removal of certain Atgs [13]. Previously, it was shown that the PI3P phosphatase Ymr1 plays a role in Atg dissociation from APs. Specifically, depletion of Ymr1 results in the accumulation of sealed APs decorated with Atgs [18]. Thus, while PI3P generation by a PI3P kinase is required for AP formation [19, 20], PI3P removal by a PI3P phosphatase from sealed APs is required for Atg dissociation, which is in turn required for AP fusion with the vacuole.

While we have shown that the Vps21 GTPase module plays a role in autophagy in yeast, it was not clear which step of the autophagy pathway this module regulates. Here, we show that the Vps21 GTPase module plays a role before the elusive step of AP closure. Moreover, we show that the Vps21-regulated step precedes Ymr1-mediated Atg dissociation in AP maturation. Finally, using double mutant analyses, we show that Vps21 functions between Ypt1-mediated AP formation and Ypt7-dependent AP fusion with the vacuole.

## Results

### Vps21 functions in autophagy downstream of Ypt1-mediated PAS formation

In yeast, autophagy can be induced either by nitrogen starvation or by addition of rapamycin [21]. We and others have previously shown that an autophagy-specific mutation in Ypt1 or depletion of its autophagy-specific GEF subunit, Trs85, results in a defect in PAS formation under normal growth conditions and when autophagy is induced by nitrogen starvation [8, 22–24]. In contrast, depletion of the Vps21 GTPase module components causes accumulation of APs under nitrogen starvation, and in most cells, AP clusters are seen near the vacuolar membranes [16]. Vps21 together with its paralog Ypt52 was proposed to have a role in autophagy based on autophagy defects in the double-deletion mutant cells when general autophagy was induced by rapamycin [15]. To check if Ypt1 and Vps21 GTPases function in the same pathway, we used double-mutant epistasis analysis. In this analysis, we took advantage of the different phenotypes of mutations in *YPT1* and *VPS21* to determine which phenotype masks the other. If Ypt1 functions upstream of Vps21 in autophagy, the phenotype of *ypt1-1 vps21Δ* double mutant cells should be similar to that of *ypt1-1* and not *vps21Δ* single mutant cells. PAS formation was determined by co-localization of two PAS/AP markers, Atg11 and Atg8, tagged with GFP and mCherry, respectively, during normal growth, and when autophagy is induced by nitrogen starvation or rapamycin, using live-cell fluorescence microscopy.

**Fig 1:**
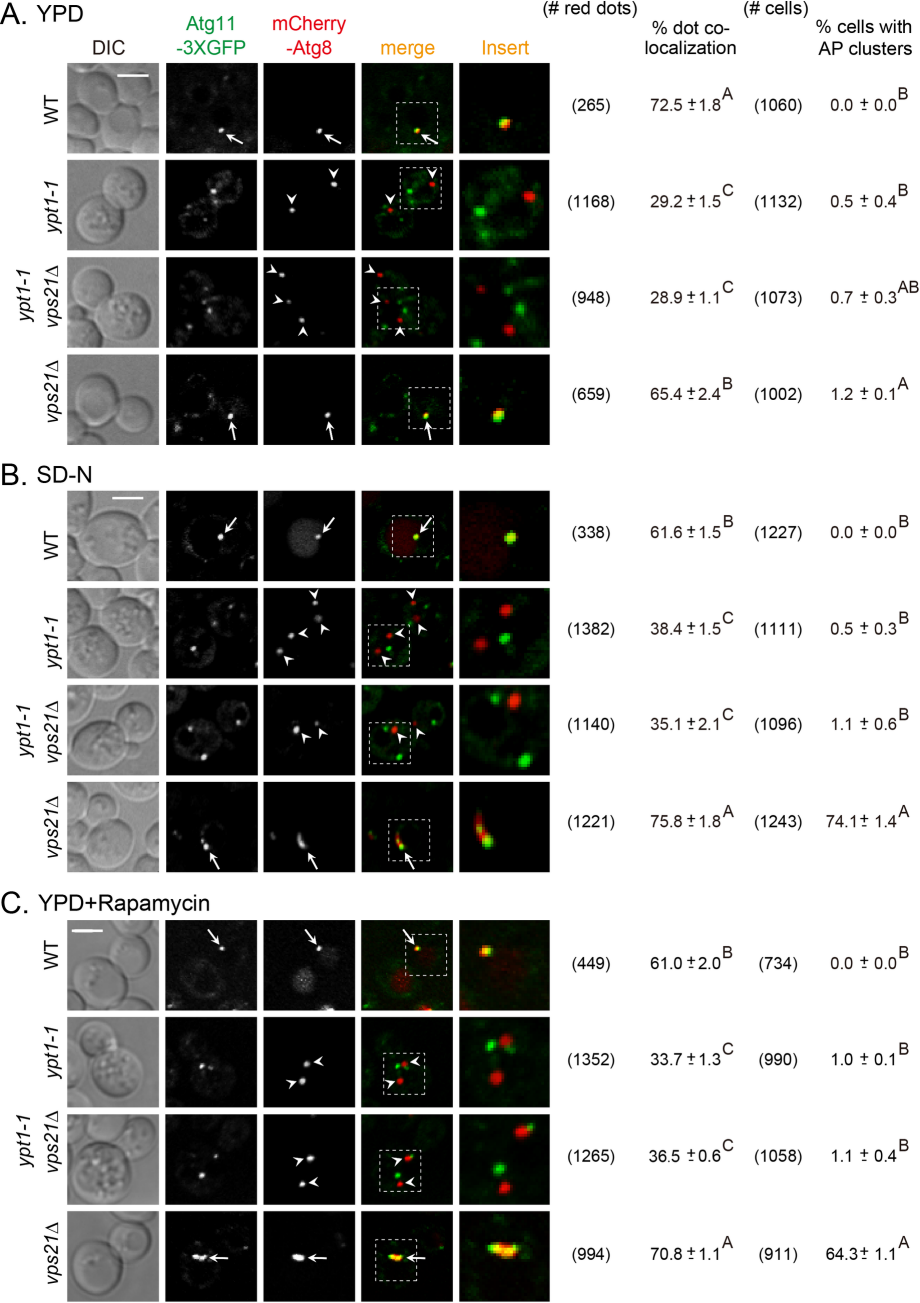
Sequential functions for Ypt1 and Vps21 in autophagy under normal growth and during autophagy. Autophagy-specific mutation in Ypt1 and depletion of Vps21 result in two distinct autophagy defects, PAS formation and AP accumulation, respectively [8, 16]. PAS or AP formation was determined by the co-localization of Atg11-3xGFP and mCherry-Atg8 in four strains: wild type (WT), *ypt1-1*, *ypt1-1 vps21Δ* and *vps21Δ* (shown from top to bottom). In each strain, the C-terminus of endogenous Atg11 was tagged with 3xGFP, and the cells were transformed with a *CEN* plasmid expressing mCherry-Atg8 (under the *CUP1* promoter). Cells grown in rich medium (YPD; **A**), were shifted to nitrogen-starvation medium (SD-N, 2 hours; **B**), and in YPD plus rapamycin (10 ng/ml, 4 hours; **C**). The co-localization of Atg11 and Atg8 was determined using live-cell fluorescence microscopy. Shown from left to right: DIC, GFP, mCherry, merge, insert, the number of red dots quantified for each strain (from 5–6 different fields), percent of dot co-localization, number of cells quantified for each strain, and percent of cells with AP clusters. Arrows indicate co-localizing puncta, arrowheads point to mCherry-Atg8 puncta that do not co-localize with Atg11; bar, 2 μm; ± represents STD, and the same capital letters of A, B, C at the right-top corner of each mean ± STD indicate no statistical significance, while different capital letters indicate significant difference (p<0.01). The *ypt1-1 vps21Δ* double mutant cells exhibit the *ypt1-1* mutant phenotype, indicating that Ypt1 functions upstream of Vps21 in the same pathway. Results in this figure represent three independent experiments.

In wild type cells, Atg8 and Atg11 co-localized to a single dot per cell (60–70% of the Atg8 dots) that represents PAS or AP. In *ypt1-1* mutant cells, both Atgs appeared as multiple dots per cell, and most of these dots did not co-localize (65–70%). In contrast, in most *vps21Δ* mutant cells, Atg11 and Atg8 co-localized on APs (65–75%) and most cells accumulate AP clusters (seen as crescents near the vacuole) when autophagy was induced. The *ypt1-1 vps21Δ* double mutant cells exhibited a phenotype similar to that of *ypt1-1*, namely, Atg8 and Atg11 appeared as multiple dots that did not co-localize (65–70%). Moreover, whereas ~65–75% of the *vps21Δ* mutant cells accumulated AP clusters when autophagy was induced by nitrogen starvation or rapamycin, no AP clusters were observed in *ypt1-1* mutant and in *ypt1-1 vps21Δ* double mutant cells (Fig 1). The mis-localization of Atg8 and Atg11 in both *ypt1-1* and *ypt1-1 vps21Δ* mutant cells was not caused by a significant decrease in protein levels (S1C and S1D Fig). These results indicate that PAS assembly is defective in *ypt1-1 vps21Δ* double mutant cells. Thus, Ypt1 functions upstream of Vps21 in both selective (normal growth) and non-selective (nitrogen starvation or rapamycin) autophagy.

The idea that the Vps21 GTPase module functions in a late step of autophagy is supported by accumulation of AP clusters in mutant cells depleted for components of this module. Additional support comes from analysis of Atg8 lipidation in these mutant cells. Atg8 lipidation is required for the attachment of Atg8 to membranes, and therefore for both AP formation and expansion [3]. The level of lipidated Atg8, Atg8-PE, was determined using immunoblot analysis in wild type, *vps21Δ* and *vps9Δ* mutant cells under nitrogen starvation. This analysis shows that similar levels of Atg8-PE are present in wild type, *vps21Δ* and *vps9Δ* mutant cells (S3 Fig). Together, the accumulation of AP clusters and the Atg8-PE level in *vps21Δ* and *vps9Δ* mutant cells indicate that the Vps21 GTPase module is not required for AP formation or expansion, but in a successive step.

### The Vps21 GTPase module functions upstream of AP closure

**Fig 2:**
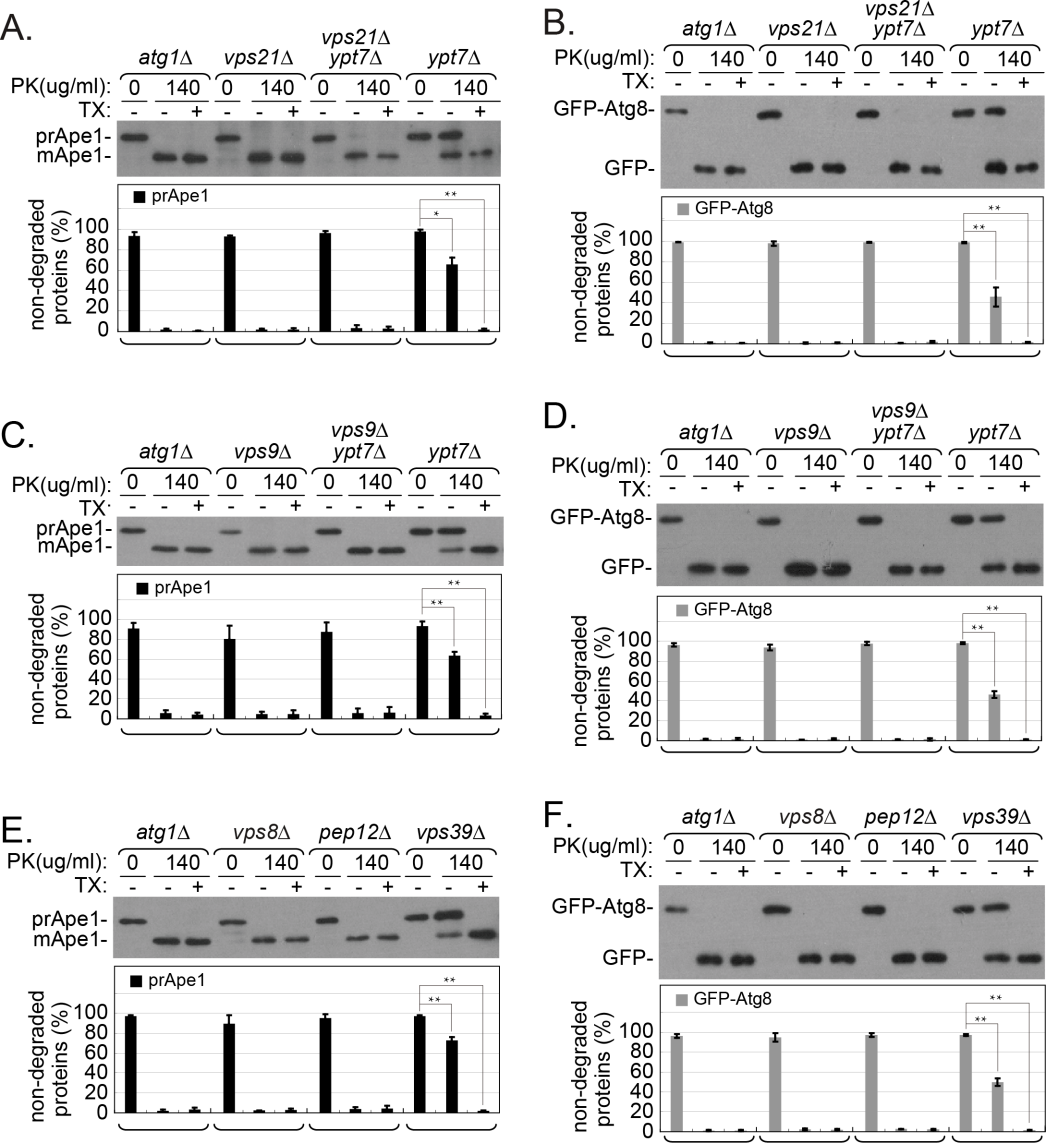
prApe1 and GFP-Atg8 are not protected from proteolysis in APs isolated from cells defective in the Vps21 module function. Protease-protection assays were performed using pellet (P5) fractions prepared from cell lysates of the indicated strains using immunoblot analysis. **A-B.** prApe1 (A) and GFP-Atg8 (B) are protected from degradation in *ypt7Δ*, but not in *vps21Δ* or *vps21Δ ypt7Δ* mutant cells. **C-D.** prApe1 (C) and GFP-Atg8 (D) are protected from degradation in *ypt7Δ*, but not in *vps9Δ* or *vps9Δ ypt7Δ* mutant cells. **E-F.** prApe1 (E) and GFP-Atg8 (F) are protected from degradation in *vps39Δ*, but not in *vps8Δ* or *pep12Δ* mutant cells. Cells expressing GFP-Atg8 from the chromosome were grown in rich medium and shifted to nitrogen-starvation medium (as in Fig 1). The P5 fractions of the cell lysates (see Materials and Methods) were treated with proteinase K (PK) with or without detergent (Triton X-100; TX). These fractions were examined by immunoblot analysis using anti-Ape1 or anti-GFP. Shown from top to bottom: strain (*atg1Δ* is used as a negative control, left), -/+ PK (0 or 140 μg/ml), -/+ TX (0 or 0.2%), Ape1 blot (A, C, E: prApe1 and mApe1), GFP blot (B, D, F: GFP-Atg8 and GFP), quantification of non-degraded proteins (%): prApe1 (black columns) and GFP-Atg8 (gray columns). For each strain, the three lanes show (from left to right): without protease (negative control), with protease (experimental), with protease and detergent (positive control, should be fully degraded). Only in cells defective in Ypt7 (A-D) or its effector Vps39 (E-F), prApe1 and GFP-Atg8 are protected from degradation by PK, whereas in all other strains, only the degradation products are seen (mApe1 and GFP) even without the addition of detergent. Columns represent mean, error bars represent STD; P values, *, p <0.05; **, p <0.01. Results in this figure represent three independent experiments.

Ypt7 is required for AP fusion with the vacuole [11]. Because the APs that accumulate in *ypt7Δ* mutant cells are sealed, the enclosed cargo is protected from degradation, as determined by an immunoblot analysis in a protease-protection assay (see Materials and Methods). Using this assay, autophagic cargo accumulating in *atg* mutant cells, which are defective in PAS and APs assembly, is sensitive to degradation [25]. Until this study, there was no mutant known to accumulate APs with cargo sensitive to degradation. This protease protection assay was used to determine whether APs that accumulate in *vps21Δ* mutant cells are sealed. Protection of two autophagy cargos was tested: prApe1 and GFP-Atg8. When not enclosed inside membranes, these cargos can be cleaved to mApe1 and GFP, respectively, by addition of proteinase K (PK) to the cell fraction (P5) that contains membrane-bound compartments. In *atg1Δ* mutant cells, in which incomplete PAS can be formed but cannot expand [26–28], the cargos were not protected, and the cleaved products are seen after addition of PK. In contrast, in *ypt7Δ* mutant cells, which accumulate APs [11], ~60% of the prApe1 and 45% of the GFP-Atg8 were protected from the protease. Importantly, the fact that the two cargos can be cleaved upon solubilization of the membranes by a detergent (Triton X-100, TX) when prepared from *ypt7Δ* mutant cells, shows that they were protected by sealed membranes. Notably, while APs accumulate in *vps21Δ* mutant cells, prApe1 and GFP-Atg8 were not protected from the protease and are cleaved (Fig 2A and 2B). This phenotype is different from that exhibited by *ypt7Δ* mutant cells, and suggests that APs accumulating in *vps21Δ* mutant cells are not sealed. Moreover, while APs accumulate in *vps21Δ ypt7Δ* double mutant cells (see below), the two autophagy cargos were also not protected from the protease (Fig 2A and 2B). Thus, as in *vps21Δ* mutant cells, in the *vps21Δ ypt7Δ* double mutant cells APs are unsealed. The fact that the *vps21Δ* phenotype masks the *ypt7Δ* phenotype indicates that Vps21 functions upstream of Ypt7 in the same pathway.

We have previously shown that other known components of the endocytic Vps21 GTPase module, the Vps9 GEF, and two of its known effectors, Vps8, a subunit of the CORVET tethering factor, and the Pep12 SNARE, are also required for autophagy, and APs accumulate upon their depletion [16]. Here, the protease protection assay was used to determine whether, as in *vps21Δ*, the cargo in APs that accumulate in *vps9Δ*, *vps8Δ* and *pep12Δ* mutant cells is accessible to PK. Both prApe1 and GFP-Atg8 were not protected from degradation in cellular membranes (P5) isolated from *vps9Δ* and *vps9Δ ypt7Δ* mutant cells (Fig 2C and 2D), indicating that, like Vps21, its Vps9 GEF is also involved in AP sealing. In addition, while autophagy cargos prApe1 and GFP-Atg8 were protected in cellular membranes isolated from mutant cells depleted for the Ypt7 effector Vps39 (70 and 50%, respectively), they were not protected in cellular membranes isolated from mutant cells depleted for the Vps21 effectors, Vps8 and Pep12 (Fig 2E and 2F). Together, these results suggest that components of the Vps21 GTPase module are required for sealing of APs, and show that this step precedes Ypt7-dependent AP fusion with the vacuole.

Using three different parameters, our previous studies provided evidence that the APs that accumulate next to the vacuolar membranes in *vps21Δ* mutant cells are outside the vacuole: 1) High magnification fluorescence microscopy; 2) EM; and 3) time-lapse microscopy which shows that individual APs move throughout the cytosol [16]. While we observed that multiple APs accumulate in clusters adjacent to the vacuolar membrane in *vps21Δ* mutant cells under autophagy-inducing conditions, Nickerson et al., proposed that some APs in *vps21Δ ypt52Δ* mutant cells are inside the vacuoles during normal growth, albeit in a minor fraction of cells (<10%) [15]. A similar observation was made in Drosophila cells depleted for Rab5(d2), which accumulate Atg8 near the lysosomal membrane, apparently inside the lysosome [29]. Additionally, it has been suggested that Rab5 plays a role in lysosomal function in murine liver cells [30]. Thus, the accumulation of APs in *vps21Δ* mutant cells could potentially happen adjacent to the vacuolar membrane inside the vacuole [15]. However, we find this unlikely because yeast cells defective in vacuolar proteases, e.g., Pep4, accumulate autophagy bodies (ABs) uniformly distributed inside their vacuoles [31]. Importantly, the AP localization and accumulation in *vps21Δ* looked different from the known vacuolar accumulation of ABs in *pep4Δ* mutant cells by either fluorescence microscopy observation or TEM (S4A and S4B Fig and [16]). Furthermore, the results from the protease protection assay show that autophagy cargos in *vps21Δ* mutant and *vps21Δ pep4Δ* double mutant cell lysates were not protected from proteases, while they were protected in *pep4Δ* mutant cell lysates (S4C and S4D Fig) to a level similar to that found in *ypt7Δ* and *vps39Δ* mutant cell lysates (Fig 2). These results imply that the accumulated APs in *vps21Δ* and *vps21Δ pep4Δ* mutant cells are unsealed and are outside the vacuole, whereas accumulated ABs in *pep4Δ* mutant cells are sealed and are inside vacuolar membranes. Taken together, the aforementioned results indicate that the function of Vps21 in autophagy precedes those of Ypt7 and Pep4, whose depletion results in AP accumulation in the cytoplasm and AB accumulation inside the vacuole, respectively.

### The Vps21 module is required for Atg dissociation from APs

**Fig 3:**
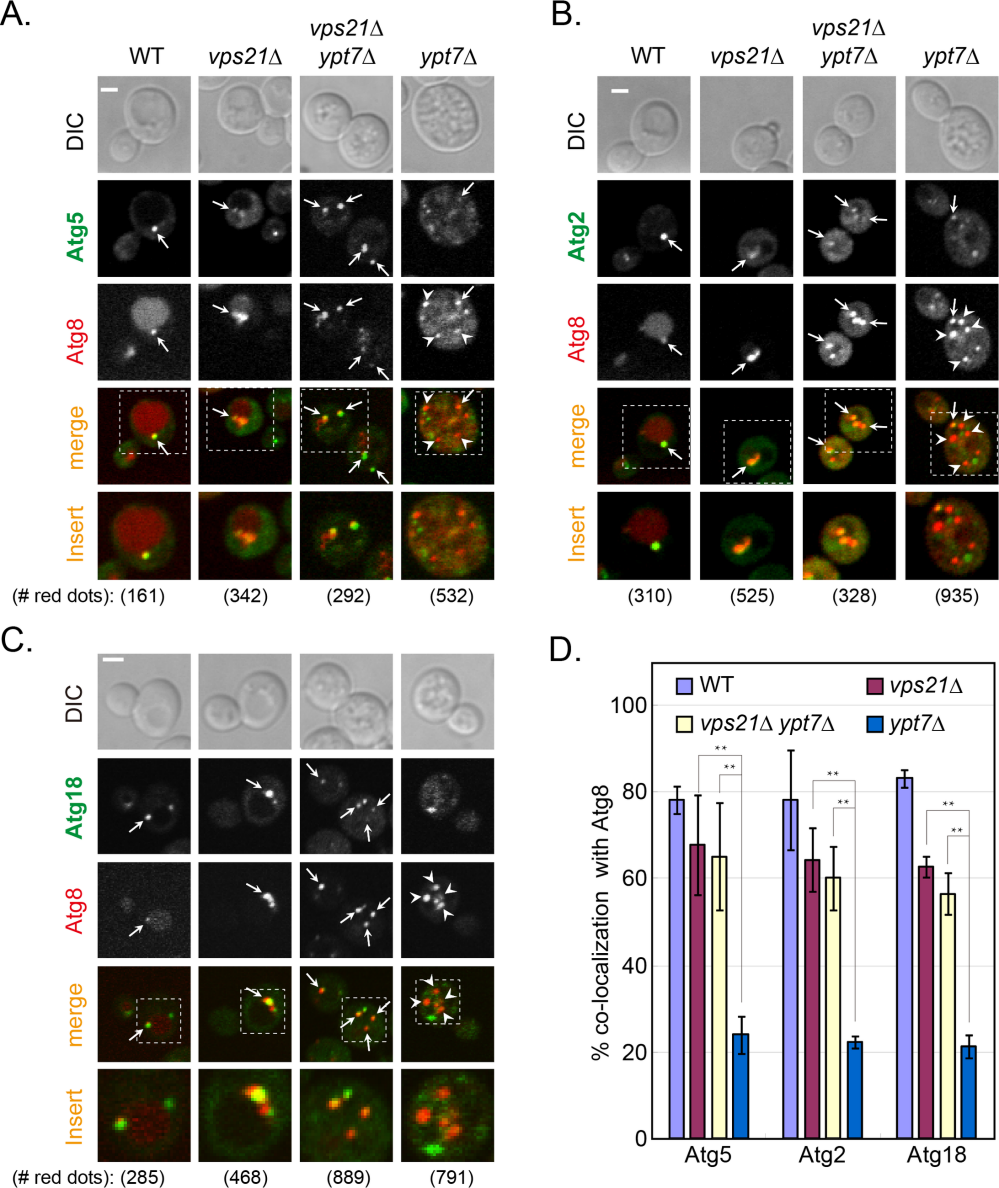
Dissociation of Atg5, Atg2 and Atg18 from accumulated APs is defective in *vps21Δ* and *vps21Δ ypt7Δ* mutant cells. Endogenous Atg5 (**A**), Atg2 (**B**), or Atg18 (**C**) were tagged with GFP at their C-terminus in four strains: WT, *vps21Δ*, *vps21Δ ypt7Δ*, and *ypt7Δ*. The cells, which also express mCherry-Atg8 (from a plasmid) as an AP marker, were grown in YPD and shifted to SD-N (as in Fig 1). The co-localization of the GFP-tagged AtgX with Atg8 was determined using live-cell fluorescence microscopy. Shown from top to bottom: DIC, GFP, mCherry, merge, insert, and the number of red puncta used for the quantification. Arrows indicate co-localizing puncta, arrowheads (in *ypt7Δ* mutant cells) point to mCherry-Atg8 puncta that do not co-localize with the other Atg; bar, 2 μm. **D.** Quantification of AtgX-Atg8 co-localization (%) from panels A-C in the four strains (strain color legend is shown on the top): Atg5 (left), Atg2 (middle), and Atg18 (right). In WT cells, a single dot per cell that contains both Atgs represents PAS or AP. Whereas Atg5, Atg2 and Atg18 are removed from most APs that accumulate in *ypt7Δ* mutant cells, they remain on most APs that accumulate in *vps21Δ* and *vps21Δ ypt7Δ* mutant cells. Columns represent mean, error bars represent STD, and P values, **, p<0.01. Results in this figure represent three independent experiments.

**Fig 4:**
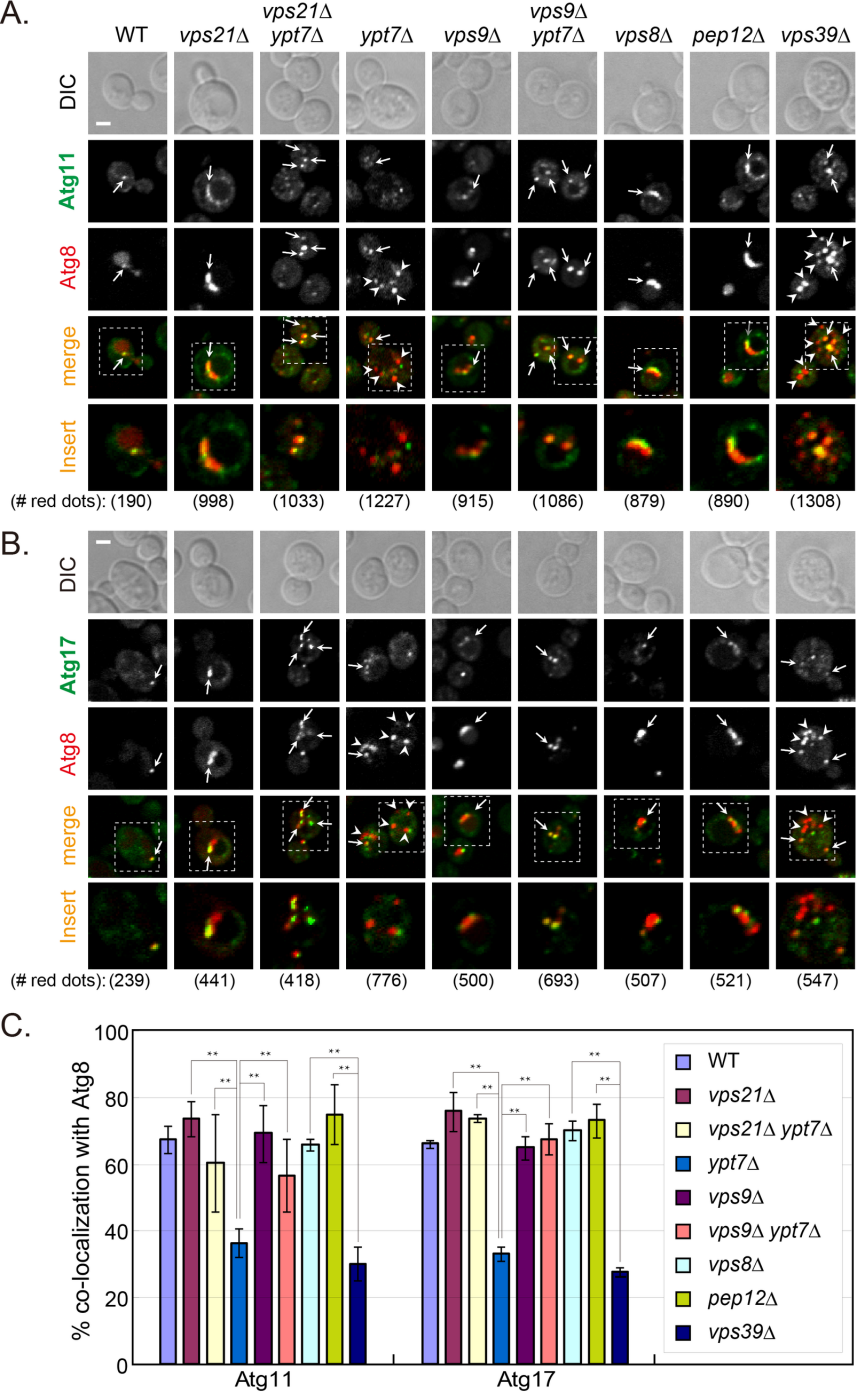
Atg11 and Atg17 dissociate from APs in cells depleted for Ypt7 and Vps39, but remain on APs in cells depleted for the Vps21 module components. Endogenous Atg11 (**A**), or Atg17 (**B**) was tagged with GFP at their C-terminus in indicated strains; from left-to-right: WT, *vps21Δ*, *vps21Δ ypt7Δ*, *ypt7Δ*, *vps9Δ*, *vps9Δ ypt7Δ*, *vps8Δ*, *pep12Δ*, and *vps39Δ*. The cells, which express mCherry-Atg8 (from a plasmid) as an AP marker, were grown in YPD and shifted to SD-N (as in Fig 1). The co-localization of the GFP-tagged AtgX with Atg8 was determined using live-cell fluorescence microscopy. Shown from top to bottom: DIC, GFP, mCherry, merge, insert, and the number of red puncta used for the quantification. Arrows indicate co-localizing puncta, arrowheads (in *ypt7Δ* and *vps39Δ* mutant cells) point to mCherry-Atg8 puncta that do not co-localize with the other Atg; bar, 2 μm. **C.** Quantification of percent AtgX-Atg8 co-localization from panels A-B in the indicated strains: Atg11 (left), and Atg17 (right), strain color legend is shown on the right. In WT cells, a single dot per cell that contains both Atgs, represents PAS or AP. Whereas Atg11 and Atg17 are removed from most APs that accumulate in *ypt7Δ* and *vps39Δ* mutant cells, they remain on most APs that accumulate in mutant cells defective in the Vps21 module as well as in the double mutant cells. Columns represent mean, error bars represent STD, and P values, **, p <0.01. Results in this figure represent at least three independent experiments.

One hallmark of AP maturation is the dissociation of several Atgs from the AP, e.g., Atg2, Atg5 and Atg18. In contrast, some Atg8 remains attached to APs as they fuse with the vacuole [13]. We wished to determine whether Atgs are still present on unsealed APs that accumulate in cells depleted for Vps21. The co-localization of Atg2, Atg5 and Atg18 with the AP marker Atg8 was determined in *vps21Δ*, *ypt7Δ*, and *vps21Δ ypt7Δ* mutant cells under nitrogen starvation. Whereas Atg2, Atg5 and Atg18 were present only on ~20% of APs that accumulate in cells depleted for Ypt7, they were present on ~60% of APs and AP clusters that accumulate in *vps21Δ* and *vps21Δ ypt7Δ* mutant cells (Fig 3). Like Atg2, Atg5 and Atg18, Atg11 and Atg17 also remained on most APs that accumulate in *vps21Δ* and *vps21Δ ypt7Δ* mutant cells (60–80%), but significantly less were present on APs in *ypt7Δ* mutant cells (~30–40%). In addition, Atg11 and Atg17 also remained on the majority of APs in *vps9Δ*, *vps9Δ ypt7Δ*, *vps8Δ* and *pep12Δ* mutant cells, but not in *vps39Δ* mutant cells (Fig 4). Together, these results show that Atg dissociation is defective in mutant cells depleted for Vps21, its Vps9 GEF or its Vps8 and Pep12 effectors, but not in cells depleted for Ypt7 or its Vps39 effector. Moreover, double mutant analyses support the idea that both Vps9 and Vps21 function upstream of Ypt7 in autophagy.

### Vps21 functions upstream of the PI3P phosphatase Ymr1 in AP maturation

The PI3P phosphatase Ymr1 was previously demonstrated to play a role in Atg dissociation during AP maturation. Specifically, upon addition of rapamycin, *ymr1Δ* exhibit a defect in general autophagy, accumulation of sealed APs in the cytoplasm, and a failure of Atg dissociation from these APs [18]. We wished to explore the relationship between the roles of Vps21 and Ymr1 in autophagy and to determine whether they function in the same pathway sequentially. This is especially important since the autophagic phenotypes of both *vps21Δ* and *ymr1Δ* are partial [16, 18]. The autophagy defects of the two single-deletion mutant strains and those of the double-deletion mutant strain, were compared upon induction of generic autophagy under nitrogen starvation. Analyses of autophagy cargos processing during nitrogen starvation show that in the single mutant cells, some of the prApe1 and GFP-Atg8 is processed to mApe1 and GFP, respectively. Whereas *ymr1Δ* mutant cells exhibit a less severe mApe1 processing defect than *vps21Δ* mutant cells, GFP-Atg8 processing is similar in both mutant cells. Importantly, the autophagy phenotypes of the *vps21Δ ymr1Δ* double mutant cells do not exceed those of the single deletion strains (S5 Fig). These results are consistent with the idea that Vps21 and Ymr1 function in the same pathway.

**Fig 5:**
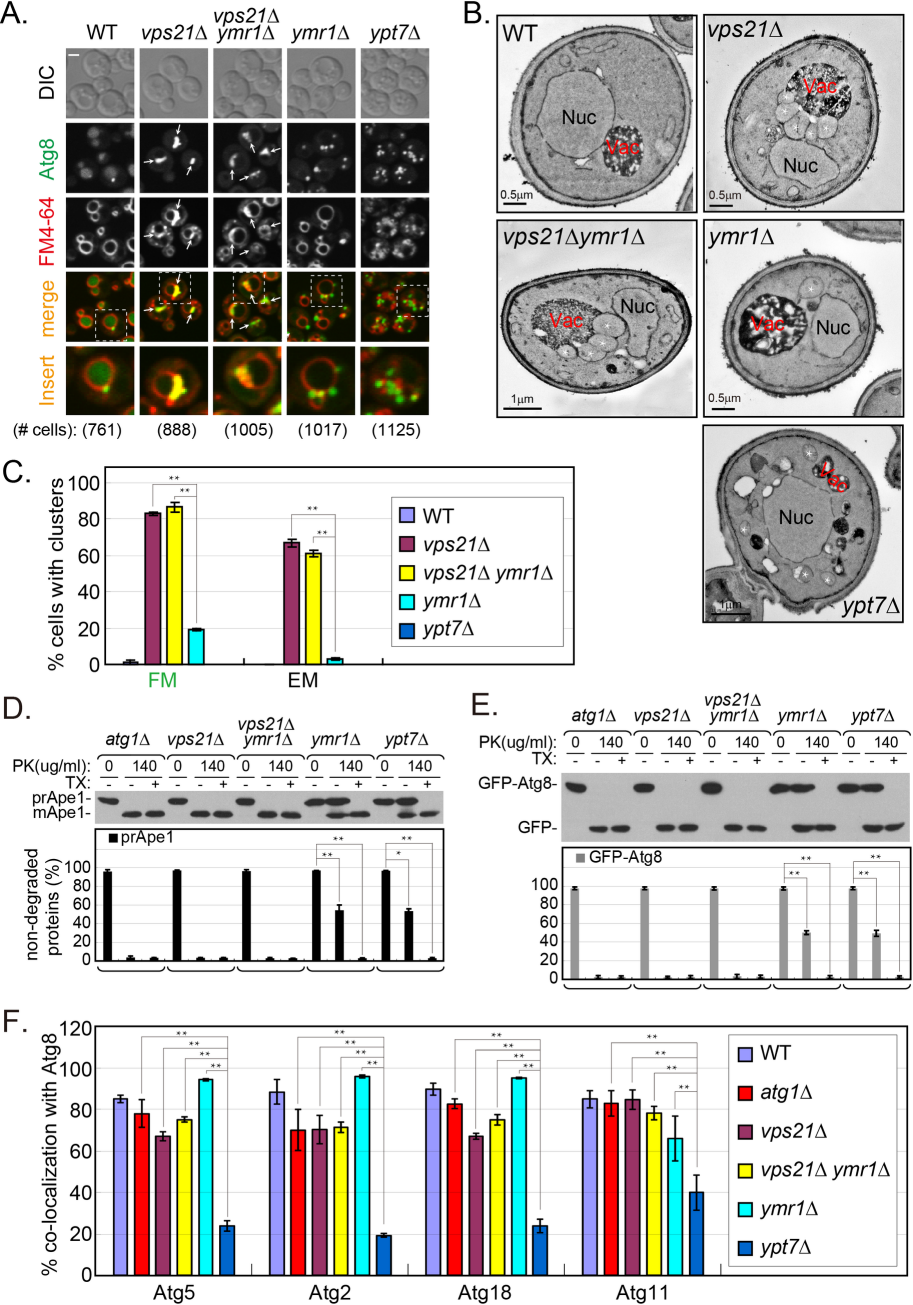
Vps21 functions upstream of Ymr1 in AP maturation. **A.** GFP-Atg8-labled APs accumulate in clusters near the vacuoles of *vps21Δ* and *vps21Δ ymr1Δ*, but not of *ymr1Δ* (or *ypt7Δ*), mutant cells. Cells expressing GFP-Atg8 from the chromosome, were grown in YPD and shifted to SD-N for 2 hours (as in Fig 1), and FM4-64 (red dye) was added for the second hour to visualize the vacuolar membrane. Cells were visualized by live-cell fluorescence microscopy (FM). Shown from left to right: WT, *vps21Δ*, *vps21Δ ymr1Δ*, *ymr1Δ* and *ypt7Δ*. Shown from top to bottom: strains, DIC, GFP, FM4-64, merge, insert, and number of cells visualized with GFP-Atg8. Arrows indicate Atg8 clusters co-localizing with FM4-64; bar, 2 μm. 80–90% of the *vps21Δ* and *vps21Δ ymr1Δ* mutant cells contain Atg8 clusters (see quantification in C). **B.** Accumulation of AP clusters outside the vacuole of *vps21Δ* and *vps21Δ ymr1Δ*, but not *ymr1Δ* or *ypt7Δ* mutant cells. The ultra-structure of cells as starved in A was visualized by electron microscopy (EM). Representative cells are shown. APs appear as clusters outside the vacuole of both *vps21Δ* and *vps21Δ ymr1Δ* mutant cells. Nuc, nucleus; Vac, vacuole; white asterisks mark individual APs. Cell slices with ≥2 neighboring APs were scored as containing an AP cluster; ~60% of the *vps21Δ* and *vps21Δ ymr1Δ* mutant cells contain AP clusters; ≥300 cells were visualized for each strain (see quantification in C). **C.** Quantification of results from A and B is shown as the percent of cells with GFP-Atg8 clusters by FM (left) and AP clusters by EM (right) in the indicated strains (strain color legend is shown on the right). Columns represent mean, and error bars represent STD. **D-E.** Whereas sealed APs accumulate in *ymr1Δ* and *ypt7Δ*, unsealed APs accumulate in *vps21Δ* and *vps21Δ ymr1Δ* mutant cells. Protease protection analysis of Ape1 blot (D) and GFP blot (E), was done (as described in Fig 2) in the following strains (from left-to-right): *atg1Δ* (unprotected control), *vps21Δ*, *vps21Δ ymr1Δ*, *ymr1Δ*, and *ypt7Δ*. Shown from top to bottom: strain, -/+ PK, -/+ TX, blot: and quantification of non-degraded proteins (prApe1, black columns; GFP-Atg8, gray columns). Both prApe1 and GFP-Atg8 are protected from degradation in AP fractions isolated from *ymr1Δ* and *ypt7Δ*, but not from *vps21Δ* and *vps21Δ ymr1Δ* mutant cells. **F.** Atg dissociation from APs is defective in *vps21Δ*, *vps21Δ ymr1Δ* and *ymr1Δ*, but not in *ypt7Δ* mutant cells (strain color legend is shown on the right). Quantification of results from S6 Fig and S7 Fig showing the percent of co-localization of AtgX with Atg8; from left to right: Atg5, Atg2 (S6 Fig), Atg18 and Atg11 (S7 Fig). Columns represent mean, error bars represent STD, and P values, **, p <0.01. Results in this figure represent three independent experiments.

We next explored AP accumulation under nitrogen starvation in cells depleted for Vps21, Ymr1, or both. We have previously shown that APs accumulate in clusters next to the vacuole in *vps21Δ* mutant cells [16]. AP accumulation was determined here using two approaches, live-cell fluorescence microscopy and electron microscopy (EM). As previously shown [18], like in *ypt7Δ*, multiple GFP-Atg8 marked APs are dispersed in the cytoplasm of *ymr1Δ* mutant cells, and only about 20% of the cells have a GFP-Atg8 cluster. Interestingly, as seen by the FM4-64 staining, unlike in *ypt7Δ*, vacuoles in *ymr1Δ* mutant cells are not fragmented. This observation indicates that dispersal of APs in the cytoplasm observed in *ymr1Δ* and *ypt7Δ* mutant cells is not caused by vacuole fragmentation. In agreement with the live-cell fluorescence microscopy analysis, AP accumulation in clusters was observed in ~60% of *vps21Δ*, compared to <5% in *ymr1Δ* mutant cells using EM. Importantly, AP cluster accumulation in *vps21Δ ymr1Δ* double mutant cells is similar to that observed in *vps21Δ*, and not in *ymr1Δ*, single mutant cells in both assays (Fig 5A–5C), supporting the idea that Vps21 functions upstream of Ymr1 in autophagy.

It was previously shown that some prApe1 is protected from proteases in APs isolated from *ymr1Δ* mutant cells, suggesting that APs that accumulate in these cells are sealed [18]. To further dissect the relationship between depletion of Vps21 and Ymr1, single and double mutant cells were tested by the protease protection assay using two cargos. As in *ypt7Δ*, ~50% of prApe1 and GFP-Atg8 was protected from the protease in *ymr1Δ* mutant cells. In contrast, in *vps21Δ ymr1Δ* double mutant cells, as in *vps21Δ*, both autophagy cargos were not protected from the protease (Fig 5D–5E). These results show that Vps21 functions prior to Ymr1.

A defect in Atg dissociation from APs that accumulate in *ymr1Δ* mutant cells was previously reported [18]. Because we show here that *vps21Δ* mutant cells are also defective in Atg dissociation from APs, we expected that if Vps21 and Ymr1 function in the same pathway, *vps21Δ ymr1Δ* double mutant cells will show a phenotype similar to and not exceeding that of the single deletion strains. Indeed, in live-cell fluorescence microscopy analyses, Atg5, Atg2, Atg18 and Atg11 were co-localized with APs and AP clusters that accumulate in *vps21Δ ymr1Δ* mutant cells (Fig 5F and S6 and S7 Figs). These results agree with the idea that Vps21-dependent AP sealing shown above, precedes Atg dissociation during AP maturation, which is defective in cells depleted for Vps21 and/or Ymr1.

**Fig 6:**
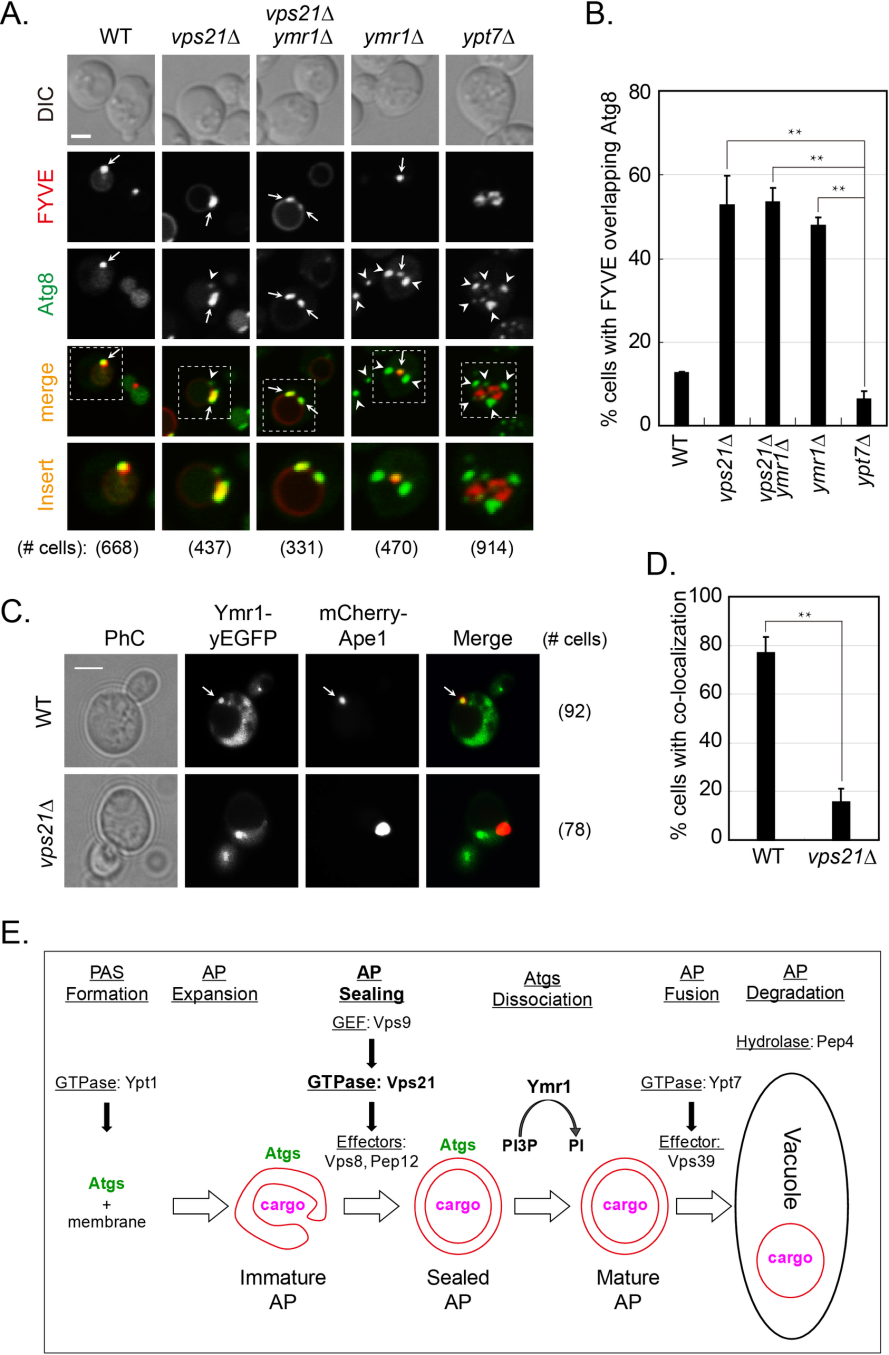
Localization of PI3P and Ymr1 to APs in wild type and mutant cells. **A-B.** PI3P is present on APs that accumulate in *vps21Δ*, *vps21Δ ymr1Δ*, and *ymr1Δ*, but not in *ypt7Δ*, mutant cells. Co-localization of the PI3P-binding FYVE domain with Atg8-marked APs (A). Indicated strains expressing endogenously tagged GFP-Atg8 and DsRed-FYVE from a 2μ plasmid, were grown in YPD and shifted to SD-N for 2 hours. Cells were visualized by live-cell fluorescence microscopy. Strains (from left to right): WT, *vps21Δ*, *vps21Δ ymr1Δ*, *ymr1Δ*, and *ypt7Δ*. Shown from top to bottom: DIC, DsRed, GFP, merge, insert, and number of cells expressing the FYVE domain used for quantification. Arrows indicate co-localizing puncta (or AP clusters), arrowheads point to GFP-Atg8 puncta that do not co-localize with the FYVE domain; bar, 2 μm. Quantification (B) of the percent of cells with FYVE domain co-localizing with Atg8-marked APs from panel A. **C-D.** Localization of Ymr1 to APs is defective in *vps21Δ* mutant cells. Ymr1-yEGFP and the AP marker mCherry-Ape1 were co-expressed in WT and *vps21Δ* mutant cells. After 2 hours of nitrogen starvation, co-localization of the two proteins was determined using live-cell fluorescence microscopy (C). Strains: WT (top) and *vps21Δ* (bottom). From left to right: PhC, GFP, mCherry, merge and number of cells used for quantification. Arrow indicates co-localizing puncta. Quantification (D) of the percent of cells with co-localizing Ymr1 and Ape1 from panel C. In B and D, columns represent mean, error bars represent STD, and P values, *, p<0.05; **, p <0.01. Results in panels A-D represent three independent experiments. **E.** Model for the regulation of three successive steps in autophagy by Ypt GTPases: Ypt1 regulates PAS formation [8]; the Rab5-like module–Vps9 GEF, Vps21, and Vps8 and Pep12 effectors–regulates AP maturation (shown here), and Ypt7 (together with its Vps39 effector) controls mature AP fusion with the lysosome [11, 13]. Moreover, the two successive steps in AP maturation are separated: Vps21 regulates AP closure directly or indirectly, and Ymr1 mediates PI3P hydrolysis and Atg removal from closed APs.

PI3P is required for early autophagy [32], and its removal in later steps was inferred from the role of the PI3P phosphatase Ymr1 after AP accumulation [18]. However, PI3P removal from APs was not shown directly in the later study. Here, we explored PI3P presence on Atg8-marked APs using the PI3P reporter DsRed-FYVE domain [33] and live-cell fluorescence microscopy. This is important since PI3P also decorates endosomes [34]. Co-localization of DsRed-FYVE domain with GFP-Atg8 showed that while PI3P was present only on ~5% of cells with APs that accumulate in *ypt7Δ* mutant cells, it was present on ~50% of cells with APs or AP clusters that accumulate in *vps21Δ*, *vps21Δ ymr1Δ*, and *ymr1Δ* mutant cells (Fig 6A and 6B). These results show that PI3P removal from APs is defective in cells depleted for Vps21 and/or Ymr1. In addition, these results agree with the idea that Vps21-dependent AP sealing shown above, also precedes Ymr1-dependent PI3P removal during AP maturation, which is defective in cells depleted for Vps21 and/or Ymr1.

The above mentioned double-mutant analyses suggest that the Vps21-dependent step precedes the Ymr1-dependent step in the autophagy pathway. Therefore, we wished to determine whether Ymr1 localization to APs is dependent on Vps21. It was previously reported that the majority (~75%) of RFP-Ape1 marked APs co-localize with GFP-tagged Ymr1 during autophagy in wild type cells [18]. We compared this co-localization in wild type and *vps21Δ* mutant cells expressing Ymr1-yEGFP and mCherry-Ape1 as an AP marker. Whereas Ymr1 co-localized with Ape1 in ~80% of wild type cells, it does so only in ~15% of *vps21Δ* mutant cells (Fig 6C and 6D). This result suggests that the localization of Ymr1 to APs is dependent on Vps21 and further supports the order of their function, which is based on double mutant analyses. Together, our results show that Vps21 and Ymr1 function sequentially in two separate steps of AP maturation: during or upstream of AP sealing, and PI3P hydrolysis-dependent Atg dissociation from closed APs, respectively. As for the order of function of Ymr1 and Ypt7, while *ymr1Δ* mutant cells accumulate dispersed APs like *ypt7Δ* mutant cells, unlike *ypt7Δ* mutant cells, they accumulate APs decorated with Atgs and PI3P.

## Discussion

AP maturation, which is required for AP fusion with the vacuole, includes membrane sealing and removal of most Atgs. Results presented here suggest that the Rab5-related Vps21, in the context of its endocytic GEF-GTPase-effector module, regulates AP closure. Specifically, while cells depleted for members of the Vps21 GTPase module accumulate APs, the cargo enclosed in these APs is not protected from degradation by proteases, implying that these APs are not sealed. AP accumulation next to, but outside of, the vacuole, is supported by microscopy and epistasis analyses showing that Vps21 functions upstream of both Ymr1 and Ypt7, which accumulate sealed APs dispersed in the cytoplasm. Currently, the evidence that APs accumulating in *vps21Δ* mutant cells are unsealed is provided by one approach, the protease protection assay. Future higher-resolution morphological analyses should be performed to confirm this idea. Notably, the observation that Atg dissociation, which follows AP closure, is also defective in mutant cells depleted for members of the Vps21 GTPase module, further supports a role of this module in AP maturation. To our knowledge, this is the first report that identifies **regulators** that function between AP expansion and closure.

In addition, using double mutant analyses, we demonstrate that Vps21 functions in autophagy downstream of Ypt1 and upstream of Ypt7. This establishes a cascade of three successive steps in autophagy regulated by Rab GTPases: Ypt1-mediated AP formation, Vps21-regulated AP closure, and Ypt7-dependent AP fusion with the vacuole (Fig 6E). Interestingly, while Ypt1 regulates AP formation in the context of an autophagy-specific module [8], the functions of Vps21 and Ypt7 in autophagy are performed together with their cognate GEFs and effectors that also mediate endocytosis [13, 16]. The possible reason for this discrepancy is that whereas Ypt1 regulates delivery of different cargos to the secretory and the autophagy pathways, Vps21 and Ypt7 regulate preparation of membranes surrounding endosomes or APs for fusion with the same organelle, the lysosome. Because in endocytosis Vps21 and Ypt7 also function sequentially, the endocytic and autophagy pathways seem to converge at the Vps21-mediated step.

Previously, the PI3P phosphatase Ymr1 was shown to regulate Atg dissociation from APs [18]. While PI3P has been shown to play a major role early in the autophagy pathway [19, 32], it can be inferred from the involvement of Ymr1 that PI3P removal from APs might be required for the completion of this pathway, specifically for Atg dissociation. Because depletion of either Vps21 or Ymr1 does not result in a complete autophagy block [16, 18], it was important to determine whether they function sequentially in the same pathway or in parallel pathways. Double mutant and localization analyses shown here establish that Vps21 and Ymr1 function sequentially in AP closure and Atg dissociation, respectively. Moreover, using a PI3P reporter, we show that PI3P is present on APs in cells depleted for Vps21 and/or Ymr1, but not for Ypt7. Together, these results demonstrate that Vps21 and Ymr1 regulate the two successive steps of AP maturation: AP closure and PI3P-dependent Atg dissociation, respectively (Fig 6E).

What could be the mechanism of Vps21-mediated AP closure? The fact that Vps21 functions in autophagy and endocytosis in the context of the same module might provide some clues. In endocytosis, Vps21 mediates the formation of late endosomes by the recruitment of the tethering complex CORVET and the Pep12 t-SNARE. Tethering factors and SNAREs mediate membrane fusion. However, it is currently unclear which fusion event is regulated by Vps21 and its effectors in endocytosis. One possibility is that they regulate the homotypic fusion of endosomes to create the large late endosome that contains multiple intra-luminal vesicles (ILVs) [10, 35]. Similarly, it is possible that in autophagy, Vps21 together with its effectors mediate homotypic fusion of APs. However, the size of APs that accumulate in *vps21Δ* mutant cells, which is within the 400–900 nm range reported for AP size [2], argues against this possibility. Another possibility is that in endocytosis Vps21 mediates maturation of early endosomes to late endosomes, which are also called multi-vesicular bodies (MVBs). In this process, the early endosomal membrane invaginates to form pouches, and sealing of these “pouches” results in formation of ILVs inside the MVBs [36]. In autophagy, while sealing of the double membrane APs does not require membrane invagination because the whole AP resembles “a single pouch”, the topology of its sealing is similar to that of ILV sealing [13]. Therefore, it is tempting to propose that Vps21, together with its effectors, CORVET (e.g., Vps8) and SNAREs (e.g., Pep12), mediate membrane scission of the open pouch to create sealed APs [37]. Formally, it is possible that the Vps21 module regulates a yet unknown step that follows AP expansion and precedes AP closure. Regardless, in both endocytosis and autophagy, subsequent progression to the Ypt7-mediated fusion of endosome or sealed AP with the lysosome is expected to follow.

In mammalian cells a number of Atgs were implicated in AP closure. First, overexpression of an inactive mutant Atg4B, which interferes with the lipidation of LC3/Atg8, results in accumulation of incomplete APs [38]. Second, excess of sphingolipids, which disturbs Atg9A trafficking, results in abnormally swollen and unclosed APs [39]. Third, using depletion analyses of LC3, GABARAP, Atg conjugation systems, and the Atg2-binding protein EPG-6, a role for these proteins was suggested in AP closure [21, 40, 41]. In all these cases, the culprit, the mammalian Atg8, Atg9, GABARAP, Atg conjugation systems, and EPG-6, plays a known role in AP formation or expansion [42], and, therefore, the effect on AP closure is not specific to this step and can be indirect. Because neither Vps21 nor Ypt7 is required for AP formation and/or expansion, their role in autophagy, e.g., AP closure and fusion, seems to be specific to a late step of autophagy, and possibly a direct role. Because these players are conserved, we expect that the role of Rab5 in AP closure will pertain to mammalian cells. Currently, while a role for Rab5 in autophagy is accepted [17], the specific step in which it was implicated varies from AP formation [43] to a late step after that regulated by Rab7 [29]. Future studies should elucidate mechanisms that underlie AP closure and reveal whether, like AP formation and fusion with the lysosome, they are conserved from yeast to humans.

## Materials and methods

### Strains, plasmids, and reagents

Yeast strains and plasmids used in this study are listed in S1 Table. All yeast and *Escherichia coli* transformations were as previously described [44].

Construction of strains used for live-cell microscopy of Atg11-3XGFP and mCherry-Atg8 in *ypt1-1* related cells: Atg11-3GFP-PG5 plasmid was linearized and integrated in SEY6210 as previously described [45]. This strain was mated with *ypt1-1* mutant cells [8] for dissection to get Atg11-3XGFP tagged wild type (WT) and *ypt1-1* mutant cells. The genotypes of the latter strain was validated by complementation of the *ypt1-1* mutant phenotype by *YPT1* (S1A and S1B Fig). *VPS21* was deleted with the hygromycin resistance cassette to get Atg11-3XGFP tagged *vps21Δ* and *ypt1-1 vps21Δ* mutant cells. Wild type and mutant strains expressing endogenously tagged Atg11-3XGFP were transformed with *CUP1*p-mCherry-Atg8-415 for examining co-localizations under growth and autophagy inducing conditions. Similarly, p1K-GFP-Atg8-406 plasmid was linearized and integrated in WT and *ypt1-1* mutant cells as previously described [45]. *VPS21* was deleted from the obtained strains using the hygromycin resistance cassette to get GFP-Atg8 tagged *vps21Δ* and *ypt1-1 vps21Δ* mutant cells.

Construction of the strains used for live-cell microscopy of Atg-GFP dissociation from mCherry-Atg8 marked APs or dsRed-FYVE on GFP-Atg8 marked APs: ORF of specific gene was deleted from AtgX-GFP (X for 5, 2, 18, 11, 17) or GFP-Atg8 tagged wild type with a drug resistance cassette (hygromycin or KanMX) or the *LYS2* gene to get different deletion mutants by PCR amplification and recombination. The AtgX-GFP tagged wild type and mutant strains were transformed with *CUP1*p-mCherry-Atg8-415 for examining co-localizations under starvation conditions. Alternatively, pRS304-mCherry-Atg8 was linearized and integrated in AtgX-GFP (X for 5, 2, 18, 11) tagged strain in SEY6210 background to get AtgX-GFP mCherry-Atg8 strains. Other mutants were obtained with deletion of ORF from AtgX-GFP mCherry-Atg8 strains as above. Finally, GFP-Atg8 tagged cells were examined with the lipophilic dye FM4-64 [45] to observe Atg8 clusters near the vacuolar membranes or with a DsRed-FYVE plasmid to observe FYVE on GFP-Atg8 marked APs.

Plasmids expressing mCherry-Ape1 (pNS1321) and Ymr1-yEGFP (pNS1603) under *ADH1* promoter were used for co-localization analysis. pNS1603 was constructed as follows: *YMR1* ORF without the stop codon was cloned in p415-yEGFP (pNS1492) using SpeI (vector)/AvrII (insert) and BspEI sites.

The antibodies and chemical reagents used in this project have been previously described [16].

### Yeast culture conditions and induction of autophagy

For complementation analyses, cells transformed with an empty vector (pRS425, 2μ, *LEU2*) or the plasmid for Ypt1 expression were grown in SD-Leu medium overnight and then spotted onto SD-Leu plates in ten-fold serial dilutions, and incubated at indicated temperatures. For live-cell fluorescence microscopy and biochemical analysis, yeast overnight cultures from YPD or selection medium (when a plasmid was used) were inoculated at ~0.03 OD_600_ to grow overnight to reach ~0.5 OD_600_ at 26°C in YPD, re-inoculated at ~0.05 OD_600_ to reach ~0.5 OD_600_. Autophagy was induced by one of two ways: Either 10 nM rapamycin was added to the YPD medium for 4 hours at 26°C [46], or the cells were washed and starved in SD-N medium (0.17% yeast nitrogen base without amino acid and ammonium sulfate with 2% glucose) at 26°C for 2 hours, as previously described [47]. When indicated, FM4-64 was added to a final concentration of 1.6 μM to stain the vacuole during the second hour before collecting the cells. A higher percent of *vps21Δ* mutant cells with GFP-Atg8 clusters were observed when the cells were grown to a lower OD_600_ and when they were re-inoculated three times before starvation (S2 Fig).

### Fluorescence microscopy observations and quantifications

Cells expressing fluorescently-tagged proteins from plasmids and/or the chromosome, or stained by FM4-64, were examined with a Nikon inverted research microscope Eclipse Ti as previously described [45] or with an UltraVIEW spinning-disk confocal scanner unit (PerkinElmer, Waltham, MA) with Z-stack of 13 stacks. More than five fields were visualized for each sample. The percentage of co-localized dots (clusters in *vps21Δ* mutant cells under autophagy-inducing conditions) is based on red dots/clusters quantified from 1–6 fields from each experiment. GFP-Atg8 clusters in different mutant cells were quantified as the percentage of GFP-Atg8 dots/clusters per vacuole. Data is presented as the mean ± standard deviation of each variable from three independent experiments. For co-localization analysis of Ymr1-yEGFP and mCherry-Ape1, WT (BY4741, NSY825) and *vps21Δ* (from the deletion library, NSY1648) yeast strains were co-transformed with two plasmids, pNS1603 and pNS1321. Transformants were grown to early log phase and then shifted to nitrogen-starvation media for 2 h as previously described [16] before being visualized by fluorescent microscopy using deconvolution Axioscope microscope.

### Protease protection assay

The protease protection assay combined with immunoblot analysis was carried out as previously described [25]. Briefly, cells expressing GFP-Atg8 were grown to log phase in YPD medium, induced for autophagy in SD-N medium, and then spheroplasted and lysed. Unbroken cells were removed by a centrifugation at 300 × *g* and the supernatant was subjected to a 5,000 x *g* spin. The 5,000 x *g*-spin pellet, P5, was re-suspended in buffer and used for proteinase K and Triton X-100 treatments. Following the protease treatment, proteins were precipitated with TCA for immunoblot analysis using anti-GFP or anti-Ape1 antibodies to determine the levels of degraded and non-degraded proteins.

### Immunoblot analysis

Immunoblot assays were conducted as previously described [45] and repeated at least three times. To separate Atg8-PE from Atg8, lysates of cells expressing HA-tagged Atg8 (or Atg8ΔR; integrated into the *URA3* locus) were examined on a 13.5% SDS-PAGE gel with 6 M urea, and run at a constant current of 25 mA; blots were probed with anti-HA as previously described [48]. Immunoblot bands were quantified using the IMAGEJ software (National Institutes of Health, USA). The percentage of non-degraded GFP-Atg8 was calculated as GFP-Atg8 / (GFP-Atg8 + GFP) ×100%. The percentage of non-degraded prApe1 was calculated as prApe1 / (prApe1 + mApe1) ×100%. The percentage of processed GFP was calculated as GFP / (GFP-Atg8 + GFP) ×100%. The percentage of mature Ape1 was calculated as mApe1 / (prApe1 + mApe1) ×100%. The protein levels of Atg11-3xGFP and GFP-Atg8 in mutant cells were adjusted to the loading control and compared to the WT levels (set as 100%). Bands from anti-G6PDH antibody were used as a loading control. The data are presented as the mean ± standard deviation of each variable from three independent experiments.

### Statistical analyses

The IBM SPSS Statisitcs was applied for significant analysis. For individual samples with ≥ three repeats, data were applied to Analysis Of Variance (ANOVA) analysis. If the data passed the Test of Homogeneity of Variances with significance (p>0.05) and ANOVA significance (p<0.01), then the data was subjected to Post Hoc Tests with least significant difference (LSD) or Duncan analysis to receive the significance of p values. Otherwise, if the data failed the Test of Homogeneity of Variances with significance (p<0.05), then the data was subjected to Post Hoc Tests with Dunnett T3 analysis to receive the p values. P values for relevant comparisons are represented as: n.s., not significant; *, p <0.05; **, p<0.01.

### Transmission electron microscopy

Cells were grown, processed and quantified for transmission electron microscopy as previously described [16].

## Supporting information

S1 Table Yeast strains and plasmids used in this study. **A.** Strains. Information about Yeast Strains: Genotype, Source and Figure numbers in which they were used. B. Plasmids. Information about Plasmids: Alias, Genotype, and Source.
(DOCX)

S1 Fig Growth phenotypes, Atg11 localization and protein level in *ypt1-1* and *vps21Δ* cells. Atg11 was tagged at its C-terminus with 3xGFP in WT and *ypt1-1* mutant cells used for Fig 1. Cells were transformed with a 2μ plasmid for over-expression of Ypt1 to validate the constructed strains (empty vector (ø) was used as a negative control). **A**. Overexpressed Ypt1 suppresses the temperature-sensitive growth phenotype of *ypt1-1* mutant cells. Cells were plated on SD-Leu plates (to select for the plasmid, 10-fold dilutions from left to right) and incubated at 30 or 37°C for 2 days. Shown from left to right: strain (WT or *ypt1-1*), plasmid (empty or Ypt1), 30°C and 37°C plates. **B**. Overexpressed Ypt1 suppresses the Atg11 localization pattern of *ypt1-1* mutant cells. Cells grown to log phase were visualized by live-cell fluorescence microscopy. Whereas Atg11 localizes to single puncta in WT cells (which represents PAS), it is present as multiple dots in *ypt1-1* mutant cells [8]. Shown from top-to-bottom: Strain, plasmid, PhC, GFP. Arrowheads point to Atg11-3XGFP; bar, 5 μm. **C**. Protein levels of endogenously-tagged Atg11-3XGFP in cells containing single and double *ypt1-1* and *vps21Δ* mutations. Cells of the four strains described in Fig 1A were cultured as described in Fig 1A legend. The Atg11-3XGFP protein level in their lysates was determined using immunoblot analysis and anti-GFP antibodies (G6PDH served as a loading control). The protein levels of Atg11-3XGFP were quantified based on loading control and compared to wild type (set as 100%). **D**. Protein levels of endogenously-tagged GFP-Atg8 in cells containing single and double *ypt1-1* and *vps21Δ* mutations. The level of GFP-Atg8 in cell lysates was determined with anti-GFP antibodies as in panel C. G6PDH served as a loading control. The density of GFP-Atg8 and GFP bands based on loading control were quantified with ImageJ and calculated as% (GFP-Atg8+GFP) to wild type. The data are presented as the mean ± standard deviation of each variable from three independent experiments. The difference of the mean for each strain compared to that of the *ypt1-1* under each growth condition is indicated at the bottoms of panels C and D. P values: n.s., not significant; *, p<0.05. Results in this supplementary figure are related to Fig 1.
(PDF)

S2 Fig Effect of growth conditions on the accumulation of Atg8 clusters during starvation of cells depleted for Vps21. Wild type (WT) and *vps21Δ* cells expressing GFP-Atg8 were grown under different conditions for observing GFP-Atg8 and FM4-64 patterns. **A.** GFP-Atg8 and FM4-64 patterns in WT and *vps21Δ* cells. A single colony was inoculated into YPD and grown to the indicated OD_600_ for three successive re-inoculations as follows: from 0.03, 0.06, and 0.12 OD_600_ to 1, 2 and 4 of OD_600_, respectively (marked: 1 ODX4; 2 ODX4; 4 ODX4, respectively). Additionally, a single colony of *vps21Δ* cells was inoculated into YPD to reach 1 OD_600_ without successive re-inoculations (marked: 1 ODX1). All cultures were then inoculated in YPD at 0.06 OD_600_ and grown for 6 hours (with rotation at 200 rpm) to reach mid-log phase, washed with water, and shifted to SD-N medium for 2 hours. FM4-64 was added during the second hour before collecting the cells. The co-localization of Atg8 and FM4-64 was determined using live-cell fluorescence microscopy. Shown from left-to-right: strains, culture conditions, PhC, GFP, FM4-64, merge, insert, and the number of cells quantified for each strain (from 3 different experiments). Arrows indicate co-localizing clusters, arrowheads point to GFP-Atg8 localizing in FM4-64 stained vacuoles; bar, 5 μm. **B.** Quantification of cells with GFP-Atg8 clusters (%) from panel A in the two strains with indicated growth conditions (bottom). A higher percent of *vps21Δ* mutant cells that contain Atg8 clusters is observed when cells were grown to a lower OD_600_ (from ~35 to 85%), and when the cells were re-inoculated three times versus once (~55 to 85%). Columns represent mean, error bars represent STD, and P values, **, p <0.01. Results in this figure represent three independent experiments and are relevant to Fig 1.
(PDF)

S3 Fig Atg8 lipidation was not disrupted in *vps21Δ* and *vps9Δ* cells. Cells deleted for *ATG8* and expressing HA-Atg8 (or HA-Atg8ΔR) from the *URA3* locus were grown and treated as in Fig 2. Immunoblot analysis was done using anti-HA antibodies. Shown from left to right: WT *atg8Δ*, *vps21Δ atg8Δ*, *vps9Δ atg8Δ*, all expressing HA-Atg8, and *atg4Δ atg8Δ* expressing HA-Atg8 or HA-Atg8ΔR. Shown from top to bottom: strain genotype, HA blot, G6PDH as a loading control and a bar graph showing the quantification of the Atg8-PE band as a percent of the total Atg8 protein. The level of Atg8-PE is similar in WT, *vps21Δ* and *vps9Δ*; *atg4Δ* serves as a negative control (with Atg8ΔR it can be lipidated even in the absence of Atg4). Bands were quantified for density and calculated as% of Atg8-PE accounted for total Atg8. P values, n.s., not significant; **, p<0.01. Experiments were repeated three times and representative blots are shown.
(PDF)

S4 Fig AP clusters that accumulate near the vacuolar membranes in *vps21Δ pep4Δ* are unclosed. **A.** GFP-Atg8-labled APs accumulate in clusters near the vacuoles of *vps21Δ* and *vps21Δ pep4Δ*, but inside the vacuoles in *pep4Δ*, mutant cells. Yeast cells were grown, starved and visualized as in Fig 5A. Shown from left to right: strains, DIC, GFP, FM4-64, merge, insert, and number of cells visualized with GFP-Atg8. Shown from top to bottom: WT, *vps21Δ*, *vps21Δ pep4Δ* and *pep4Δ*. Arrows indicate co-localizing Atg8 and FM4-64 clusters; bar, 2 μm. 80–90% of the *vps21Δ* and *vps21Δ pep4Δ* mutant cells contain Atg8 clusters (see quantification in B). **B.** Quantification of results from A is shown as the percent of cells with GFP-Atg8 clusters in the indicated strains. Columns represent the mean, and error bars represent STD. **C-D.** Whereas sealed APs accumulate in *pep4Δ*, unsealed APs accumulate in *vps21Δ* and *vps21Δ pep4Δ* mutant cells. Protease protection analysis was done (as described in Fig 2) in the following strains (from left to right): *atg1Δ* (unprotected control), *vps21Δ*, *vps21Δ pep4Δ*, and *pep4Δ*. Shown from top to bottom: strain, -/+ PK, -/+ TX, Ape1 blot (C) or GFP blot (D), and quantification of non-degraded proteins (prApe1, black columns in C; GFP-Atg8, gray columns in D). Both prApe1 and GFP-Atg8 are protected from degradation in AP fractions isolated from *pep4Δ*, but not from *vps21Δ* and *vps21Δ pep4Δ* mutant cells. P values, **, p <0.01. Results in this figure represent three independent experiments.
(PDF)

S5 Fig Processing of GFP-Atg8 and prApe1 under starvation is more defective in *vps21Δ* and *vps21Δ ymr1Δ* than in *ymr1Δ* mutant cells. **A.** Cells expressing GFP-Atg8 from the chromosome were grown in YPD and shifted to SD-N for the indicated time. Processing of prApe1 to mApe1 and GFP-Atg8 to GFP was determined in lysates using immunoblot analysis with anti-GFP and anti-Ape1 antibodies, respectively. Shown from top to bottom: strain, time of starvation (0 to 8 hours), Ape1 blot, GFP blot, and G6PDH blot (loading control). **B**. Quantification of the percent of processed proteins from A is shown for mApe1 from prApe1 (left), and GFP from GFP-Atg8 (right), in WT (open squares), *vps21Δ* (closed circles), *vps21Δ ymr1Δ* (closed triangles), and *ymr1Δ* (open circles), mutant cells. Points on the graphs represent mean, error bars represent STD and P values for SD-N for 8 hours: n.s., not significant; **, p <0.01. Results in this figure represent three independent experiments and complement Fig 5.
(PDF)

S6 Fig Atg5 and Atg2 dissociation from APs is defective in *vps21Δ*, *vps21Δ ymr1Δ* and *ymr1Δ*, but not in *ypt7Δ* mutant cells. The co-localization of Atg5 (**A**), and Atg2 (**B**), with the AP marker mCherry-Atg8 was determined and presented as described in Figs 3 and 4; bar, 2 μm. Results in this figure represent three independent experiments and their quantification of results from this figure is shown in Fig 5F.
(PDF)

S7 Fig Atg18 and Atg11 dissociation from APs is defective in *vps21Δ*, *vps21Δ ymr1Δ* and *ymr1Δ*, but not in *ypt7Δ* mutant cells. The co-localization of Atg18 (**A**), and Atg11 (**B**), with the AP marker mCherry-Atg8 was determined and presented as described in Figs 3 and 4; bar, 2 μm. Results in this figure represent three independent experiments and their quantification of results from this figure is shown in Fig 5F.
(PDF)
