# Supplementary material for: A Rab5 GTPase module is important for autophagosome closure
Source: PLoS Genet. 2017 Sep 21;13(9):e1007020. doi: 10.1371/journal.pgen.1007020 (PMC5626503; doi:10.1371/journal.pgen.1007020)
Supplement: S1 Table — A. Strains. Information about Yeast Strains: Genotype, Source and Figure numbers in which they were used. B. Plasmids. Information about Plasmids: Alias, Genotype, and Source. (DOCX) [file pgen.1007020.s001.docx]

**S1 Table. Yeast strains and plasmids used in this study**

1. **Strains**

Strain Genotype Source Figures

| YLY5286 | *Mat alpha leu2 trp1 ura3 lys2 ATG11-3×GFP::URA3* | This study | Fig. 1, S1A-C |
| --- | --- | --- | --- |
| YLY5287 | *Mat alpha ypt1-1 leu2 trp1 ura3 lys2 ATG11-3×GFP::URA3* | This study | Fig. 1, S1A-C |
| YLY5288 | *Mat alpha leu2 trp1 ura3 lys2 vps21∆::Hyg ATG11-3×GFP::URA3* | This study | Fig. 1, S1C |
| YLY5289 | *Mat alpha ypt1-1 leu2 trp1 ura3 lys2 vps21∆::HYG ATG11-3×GFP::URA3* | This study | Fig. 1, S1C |
| YLY5282 | *Mat alpha leu2 trp1 ura3 lys2 GFP-Atg8::URA3* | This study | Fig. S1D |
| YLY5283 | *Mat alpha leu2 trp1 ura3 lys2 ypt1-1 GFP-Atg8::URA3* | This study | Fig. S1D |
| YLY5284 | *Mat alpha leu2 trp1 ura3 lys2 vps21∆::Hyg GFP-Atg8::URA3* | This study | Fig. S1D |
| YLY5285 | *Mat alpha leu2 trp1 ura3 lys2 ypt1-1 vps21∆::Hyg GFP-Atg8::URA3* | This study | Fig. S1D |
| YLY2422 | SEY6210 *ura3::GFP-ATG8-URA3* | [[1](#_ENREF_1)] | Fig. 5A-C, S2A-B, S4, S5, S6 |
| YLY5928 | YLY2422 *atg1∆::KAN* | [[1](#_ENREF_1)] | Fig. 2, 5D-E, S4, S5C-D |
| YLY1628 | YLY2422 *vps21∆::KanMX* | This study | Fig. 2A-B, 5D-E, S4, S6 |
| YLY3889 | YLY2422 *ypt7∆::Hyg* | [[1](#_ENREF_1)] | Fig. 2A-D, 5A, 5D-E, S4 |
| YLY1630 | YLY2422 *vps21∆::KanMX ypt7∆::Hyg* | This study | Fig. 2A-B, S4 |
| YLY1643 | YLY2422 *vps9∆::KanMX* | This study | Fig. 2C-D |
| YLY5960 | YLY2422 *vps9∆::KanMX ypt7∆::Hyg* | This study | Fig. 2C-D |
| YLY3772 | YLY2422 *vps8∆::KanMX* | [[1](#_ENREF_1)] | Fig. 2E-F |
| YLY5324 | YLY2422 *pep12∆::Hyg* | [[1](#_ENREF_1)] | Fig. 2E-F |
| YLY3735 | YLY2422 *vps39∆::KanMX* | [[1](#_ENREF_1)] | Fig. 2E-F |
| YLY5037 | TN124, *atg8*Δ*::KAN ura3::3HA-ATG8-URA3* | [[2](#_ENREF_2)] | Fig. S3 |
| YLY5038 | TN124, *atg4*Δ *atg8*Δ *ura3::3HA-ATG8-URA3* | [[2](#_ENREF_2)] | Fig. S3 |
| YLY5039 | TN124, *atg4*Δ *atg8*Δ *ura3::3HA-ATG8*Δ*R-URA3* | [[2](#_ENREF_2)] | Fig. S3 |
| YLY5050 | YLY5037, *vps21*Δ*::Hyg* | This study | Fig. S3 |
| YLY5052 | YLY5037, *vps9*Δ*::Hyg* | This study | Fig. S3 |
| YLY6709 | *Mat a his4 ura3 leu2 lys2 bar1-1 RS307::LYS2 ATG5::ATG5-3×GFP-URA3* [pRS415-*CUP1p*-Cherry-Atg8] | This study | Fig. 3A, 3D |
| YLY6818 | *Mat a his4 ura3 leu2 lys2 bar1-1 RS307::LYS2 ATG5::ATG5-3×GFP-URA3 vps21*Δ*::KanMX* [pRS415-*CUP1p*-Cherry-Atg8] | This study | Fig. 3A, 3D |
| YLY6850 | *Mat a his4 ura3 leu2 lys2 bar1-1 RS307::LYS2 ATG5::ATG5-3×GFP-URA3 ypt7*Δ*::Hyg vps21*Δ*::KanMX* [pRS415-*CUP1p*-Cherry-Atg8] | This study | Fig. 3A, 3D |
| YLY6710 | *Mat a his4 ura3 leu2 lys2 bar1-1 RS307::LYS2 ATG5::ATG5-3×GFP-URA3 ypt7*Δ*::Hyg* [pRS415-*CUP1p*-Cherry-Atg8] | This study | Fig. 3A, 3D |
| YLY6634 | SEY6210 *ATG2-GFP::HIS5* | [[3](#_ENREF_3)] | Fig. 3B, 3D |
| YLY7020 | *ATG2-GFP::HIS5* [pRS415-*CUP1p*-Cherry-Atg8] | This study | Fig. 3B, 3D |
| YLY7021 | *ATG2-GFP::HIS5 vps21*Δ*::KanMX* [pRS415-*CUP1p*-Cherry-Atg8] | This study | Fig. 3B, 3D |
| YLY7022 | *ATG2-GFP::HIS5 ypt7*Δ*::Hyg vps21*Δ*::KanMX* [pRS415-*CUP1p*-Cherry-Atg8] | This study | Fig. 3B, 3D |
| YLY7023 | *ATG2-GFP::HIS5 ypt7*Δ*::Hyg* [pRS415-*CUP1p*-Cherry-Atg8] | This study | Fig. 3B, 3D |
| YLY6642 | SEY6210, *Atg18- GFP::HIS5* | [[3](#_ENREF_3)] | Fig. 3C, 3D |
| YLY7028 | *Atg18- GFP::HIS5* [pRS415-*CUP1p*-Cherry-Atg8] | This study | Fig. 3C, 3D |
| YLY7029 | *Atg18- GFP::HIS5 vps21*Δ*::KanMX* [pRS415-*CUP1p*-Cherry-Atg8] | This study | Fig. 3C, 3D |
| YLY7030 | *Atg18- GFP::HIS5 ypt7*Δ*::Hyg vps21*Δ*::KanMX* [pRS415-*CUP1p*-Cherry-Atg8] | This study | Fig. 3C, 3D |
| YLY7031 | *Atg18- GFP::HIS5 ypt7*Δ*::Hyg* [pRS415-*CUP1p*-Cherry-Atg8] | This study | Fig. 3C, 3D |
| YLY6713 | *ATG11::ATG11-3×GFP-URA3* [pRS415-*CUP1p*-Cherry-Atg8] | This study | Fig. 4A, 4C |
| YLY6820 | *ATG11::ATG11-3×GFP-URA3 vps21*Δ*::KanMX* [pRS415-*CUP1p*-Cherry-Atg8] | This study | Fig. 4A, 4C |
| YLY6852 | *ATG11::ATG11-3×GFP-URA3 ypt7*Δ*::Hyg vps21*Δ*::KanMX* [pRS415-*CUP1p*-Cherry-Atg8] | This study | Fig. 4A, 4C |
| YLY6714 | *ATG11::ATG11-3×GFP-URA3 ypt7*Δ*::Hyg* [pRS415-*CUP1p*-Cherry-Atg8] | This study | Fig. 4A, 4C |
| YLY6927 | *ATG11::ATG11-3×GFP-URA3 vps9*Δ*::KanMX* [pRS415-*CUP1p*-Cherry-Atg8] | This study | Fig. 4A, 4C |
| YLY6928 | *ATG11::ATG11-3×GFP-URA3 ypt7*Δ*::Hyg vps9*Δ*::KanMX* [pRS415-*CUP1p*-Cherry-Atg8] | This study | Fig. 4A, 4C |
| YLY6839 | *ATG11::ATG11-3×GFP-URA3 vps8*Δ*::KanMX* [pRS415-*CUP1p*-Cherry-Atg8] | This study | Fig. 4A, 4C |
| YLY6858 | *ATG11::ATG11-3×GFP-URA3 pep12*Δ*::Hyg* [pRS415-*CUP1p*-Cherry-Atg8] | This study | Fig. 4A, 4C |
| YLY6843 | *ATG11::ATG11-3×GFP-URA3 vps39*Δ*::KanMX* [pRS415-*CUP1p*-Cherry-Atg8] | This study | Fig. 4A, 4C |
| YLY6715 | *ATG17::ATG17-3×GFP-URA3* [pRS415-*CUP1p*-Cherry-Atg8] | This study | Fig. 4B, 4C |
| YLY6821 | *ATG17::ATG17-3×GFP-URA3 vps21*Δ*::KanMX* [pRS415-*CUP1p*-Cherry-Atg8] | This study | Fig. 4B, 4C |
| YLY6853 | *ATG17::ATG17-3×GFP-URA3 ypt7*Δ*::Hyg vps21*Δ*::KanMX* [pRS415-*CUP1p*-Cherry-Atg8] | This study | Fig. 4B, 4C |
| YLY6716 | *ATG17::ATG17-3×GFP-URA3 ypt7*Δ*::Hyg* [pRS415-*CUP1p*-Cherry-Atg8] | This study | Fig. 4B, 4C |
| YLY6929 | *ATG17::ATG17-3×GFP-URA3 vps9*Δ*::KanMX* [pRS415-*CUP1p*-Cherry-Atg8] | This study | Fig. 4B, 4C |
| YLY6930 | *ATG17::ATG17-3×GFP-URA3 ypt7*Δ*::Hyg vps9*Δ*::KanMX* [pRS415-*CUP1p*-Cherry-Atg8] | This study | Fig. 4B, 4C |
| YLY6840 | *ATG17::ATG17-3×GFP-URA3 vps8*Δ*::KanMX* [pRS415-*CUP1p*-Cherry-Atg8] | This study | Fig. 4B, 4C |
| YLY6859 | *ATG17::ATG17-3×GFP-URA3 pep12*Δ*::Hyg* [pRS415-*CUP1p*-Cherry-Atg8] | This study | Fig. 4B, 4C |
| YLY6860 | *ATG17::ATG17-3**×GFP-URA3 vps39*Δ*::KanMX* [pRS415-*CUP1p*-Cherry-Atg8] | This study | Fig. 4B, 4C |
| YLY3086 | YLY2422 *vps21∆::LYS2* | [[1](#_ENREF_1)] | Fig. 5A-E, S2, S5, S6 |
| YLY5322 | YLY2422 *vps21∆::LYS2 ymr1∆::Hyg* | This study | Fig. 5A-E, S6 |
| YLY5321 | YLY2422 *ymr1∆::Hyg* | This study | Fig. 5A-E, S6 |
| YLY3287 | YLY2422, *pep4*Δ*::Hyg* | This study | Fig. S5 |
| YLY3288 | YLY3086, *pep4*Δ*::Hyg* | This study | Fig. S5 |
| YLY8442 | SEY6210 *Atg5-GFP::KanMX* ATG8::*mCherry-Atg8-TRP1* | This study | Fig. 5F, S7A |
| YLY8930 | YLY8442 *atg1∆::Hyg* | This study | Fig. 5F, S7A |
| YLY8902 | YLY8442 *vps21∆::LYS2* | This study | Fig. 5F, S7A |
| YLY8880 | YLY8442 *vps21∆::LYS2* *ymr1∆::Hyg* | This study | Fig. 5F, S7A |
| YLY8729 | YLY8442 *ymr1∆::Hyg* | This study | Fig. 5F, S7A |
| YLY9023 | YLY8442 *ypt7∆::Hyg* | This study | Fig. 5F, S7A |
| YLY8500 | SEY6210 *Atg2-GFP::KanMX* ATG8::*mCherry-Atg8-TRP1* | This study | Fig. 5F, S7B |
| YLY8929 | YLY8500 *atg1∆::Hyg* | This study | Fig. 5F, S7B |
| YLY8922 | YLY8500 *vps21∆::LYS2* | This study | Fig. 5F, S7B |
| YLY8984 | YLY8500 *vps21∆::LYS2* *ymr1∆::Hyg* | This study | Fig. 5F, S7B |
| YLY8810 | YLY8500 *ymr1∆::Hyg* | This study | Fig. 5F, S7B |
| YLY9022 | YLY8500 *ypt7∆::Hyg* | This study | Fig. 5F, S7B |
| YLY8499 | SEY6210 *Atg8::mCherry-Atg8-TRP1 Atg18-GFP-URA3* | This study | Fig. 5F, S8A |
| YLY8929 | YLY8499 *atg1∆::Hyg* | This study | Fig. 5F, S8A |
| YLY8923 | YLY8499 *vps21∆::LYS2* | This study | Fig. 5F, S8A |
| YLY9053 | YLY8499 *vps21∆::LYS2* *ymr1∆::Hyg* | This study | Fig. 5F, S8A |
| YLY8811 | YLY8499 *ymr1∆::Hyg* | This study | Fig. 5F, S8A |
| YLY8672 | YLY8499 *ypt7∆::Hyg* | This study | Fig. 5F, S8A |
| YLY8470 | SEY6210 *Atg8::mCherry-Atg8-TRP1 Atg11-3GFP-URA3* | This study | Fig. 5F, S8B |
| YLY8888 | YLY8470 *atg1∆::Kan* | This study | Fig. 5F, S8B |
| YLY8668 | YLY8470 *vps21∆::LYS2* | This study | Fig. 5F, S8B |
| YLY8855 | YLY8470 *vps21∆::LYS2* *ymr1∆::Hyg* | This study | Fig. 5F, S8B |
| YLY8854 | YLY8470 *ymr1∆::Hyg* | This study | Fig. 5F, S8B |
| YLY8610 | YLY8470 *ypt7∆::Hyg* | This study | Fig. 5F, S8B |
| YLY550 | YLY2422 [pRS425-*MET3p*-  DsRed-FYVE] | This study | Fig. 6A, 6B |
| YLY552 | YLY2422 *vps21∆::LYS2* [pRS425-*MET3p*-  DsRed-FYVE] | This study | Fig. 6A, 6B |
| YLY8557 | YLY2422 *vps21∆::LYS2 ymr1**∆::Hyg* [pRS425-*MET3p*-  DsRed-FYVE] | This study | Fig. 6A, 6B |
| YLY556 | YLY2422 *ymr1∆::Hyg* [pRS425-*MET3p*-  DsRed-FYVE] | This study | Fig. 6A, 6B |
| YLY8554 | YLY2422 *ypt7∆::Hyg* [pRS425-*MET3p*-  DsRed-FYVE] | This study | Fig. 6A, 6B |
| NSY825 | BY4741, *MATa* *his3-1* *leu2∆ met15∆ ura3∆*  *WT for yeast deletion library* | [[4](#_ENREF_4)] | Fig. 6C-D |
| NSY1648 | BY4741, *vps21∆::KanMX* | This study | Fig. 6C-D |

1. **Plasmids**

Plasmid Alias Genotype Source

| pYL103 | pRS425 | | 2µ, *LEU2,* Amp | | | [[5](#_ENREF_5)] |
| --- | --- | --- | --- | --- | --- | --- |
| pYL317 |  | | pRS425-*YPT1* | | | [[6](#_ENREF_6)] |
| pYL542 | Atg5-3XGFP | | Atg5-3GFP-PG5 | | | This study |
| pYL303 | Atg11-3XGFP | | Atg11-3GFP-PG5 | | | This study |
| pYL306 | Atg17-3XGFP | | Atg17-3GFP-PG5 | | | This study |
| pYL882 | GFP | | pFA6a-GFP-kanMX6 | | | [[7](#_ENREF_7)] |
| pYL666 | mCherry-Atg8 | | pRS415-*CUP1p*-mCherry-Atg8 | | | [[3](#_ENREF_3)] |
| pYL1108 | yEmCherry-Atg8 | | pRS304-yEmCherry-Atg8 | | | [[8](#_ENREF_8)] |
| pYL818 | DsRed-FYVE | | pRS425-*MET3p*-  DsRed-FYVE | | | [[9](#_ENREF_9)] |
| pNS1321 | mCherry-Ape1 | | pRS416-*ADH1*p-mCherry-Ape1-*CYC1* terminator | | | {Lipatova, 2016 #2379} |
| pNS1603 | Ymr1-yEGFP | | pRS415-*ADH1*p- Ymr1-yEGFP-*CYC1* terminator | | | This study |
|  | |  | |  |  | |

**References**

1. Chen, Y., et al., *A Vps21 endocytic module regulates autophagy.* Mol Biol Cell, 2014. **25**(20): p. 3166-77.

2. Yu, Z.Q., et al., *Dual roles of Atg8-PE deconjugation by Atg4 in autophagy.* Autophagy, 2012. **8**(6): p. 883-92.

3. Cebollero, E., et al., *Phosphatidylinositol-3-phosphate clearance plays a key role in autophagosome completion.* Curr Biol, 2012. **22**(17): p. 1545-53.

4. Liang, Y., et al., *The role of Trs65 in the Ypt/Rab guanine nucleotide exchange factor function of the TRAPP II complex.* Mol Biol Cell, 2007. **18**(7): p. 2533-41.

5. Sikorski, R.S. and P. Hieter, *A system of shuttle vectors and yeast host strains designed for efficient manipulation of DNA in Saccharomyces cerevisiae.* Genetics, 1989. **122**(1): p. 19-27.

6. Zou, S., et al., *Trs130 participates in autophagy through GTPases Ypt31/32 in Saccharomyces cerevisiae.* Traffic, 2013. **14**(2): p. 233-46.

7. Longtine, M.S., et al., *Additional modules for versatile and economical PCR-based gene deletion and modification in Saccharomyces cerevisiae.* Yeast, 1998. **14**(10): p. 953-61.

8. Graef, M., et al., *ER exit sites are physical and functional core autophagosome biogenesis components.* Mol Biol Cell, 2013. **24**(18): p. 2918-31.

9. Katzmann, D.J., et al., *Vps27 recruits ESCRT machinery to endosomes during MVB sorting.* J Cell Biol, 2003. **162**(3): p. 413-23.
