# Supplementary figures and images for: A Rab5 GTPase module is important for autophagosome closure
Source: PLoS Genet. 2017 Sep 21;13(9):e1007020. doi: 10.1371/journal.pgen.1007020 (PMC5626503; doi:10.1371/journal.pgen.1007020)

# Figure S1

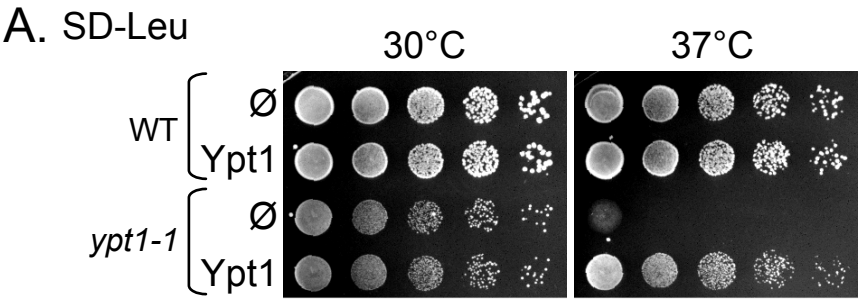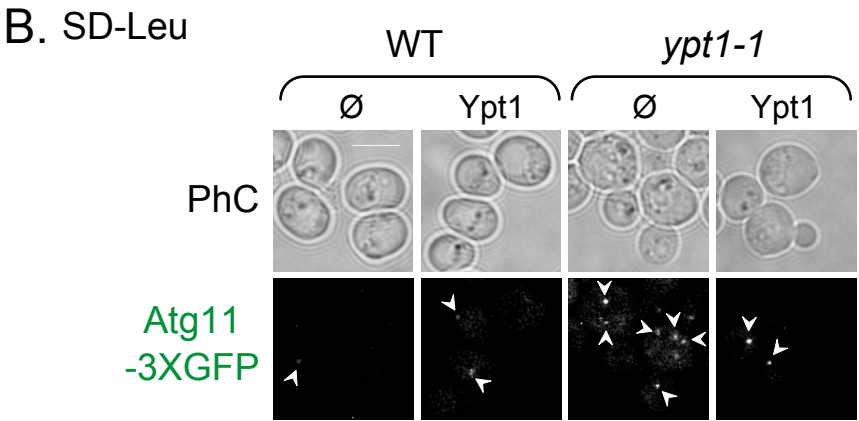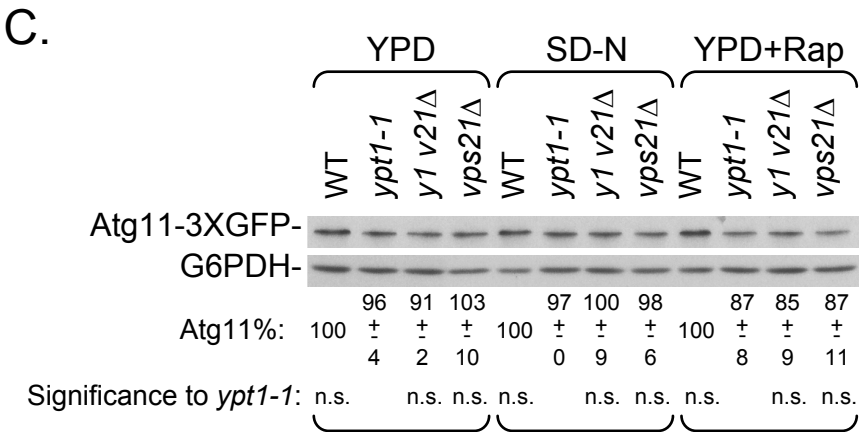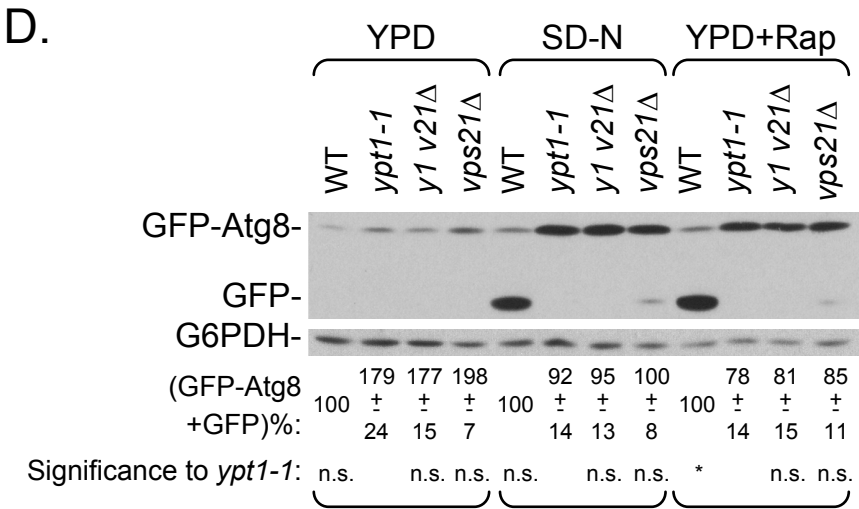

Supplement: S1 Fig — Atg11 was tagged at its C-terminus with 3xGFP in WT and ypt1-1 mutant cells used for Fig 1. Cells were transformed with a 2μ plasmid for over-expression of Ypt1 to validate the constructed strains (empty vector (ø) was used as a negative control). A. Overexpressed Ypt1 suppresses the temperature-sensitive growth phenotype of ypt1-1 mutant cells. Cells were plated on SD-Leu plates (to select for the plasmid, 10-fold dilutions from left to right) and incubated at 30 or 37°C for 2 days. Shown from left to right: strain (WT or ypt1-1), plasmid (empty or Ypt1), 30°C and 37°C plates. B. Overexpressed Ypt1 suppresses the Atg11 localization pattern of ypt1-1 mutant cells. Cells grown to log phase were visualized by live-cell fluorescence microscopy. Whereas Atg11 localizes to single puncta in WT cells (which represents PAS), it is present as multiple dots in ypt1-1 mutant cells [8]. Shown from top-to-bottom: Strain, plasmid, PhC, GFP. Arrowheads point to Atg11-3XGFP; bar, 5 μm. C. Protein levels of endogenously-tagged Atg11-3XGFP in cells containing single and double ypt1-1 and vps21Δ mutations. Cells of the four strains described in Fig 1A were cultured as described in Fig 1A legend. The Atg11-3XGFP protein level in their lysates was determined using immunoblot analysis and anti-GFP antibodies (G6PDH served as a loading control). The protein levels of Atg11-3XGFP were quantified based on loading control and compared to wild type (set as 100%). D. Protein levels of endogenously-tagged GFP-Atg8 in cells containing single and double ypt1-1 and vps21Δ mutations. The level of GFP-Atg8 in cell lysates was determined with anti-GFP antibodies as in panel C. G6PDH served as a loading control. The density of GFP-Atg8 and GFP bands based on loading control were quantified with ImageJ and calculated as% (GFP-Atg8+GFP) to wild type. The data are presented as the mean ± standard deviation of each variable from three independent experiments. The difference of the mean for each strain com [file pgen.1007020.s002.pdf]

Figure S2

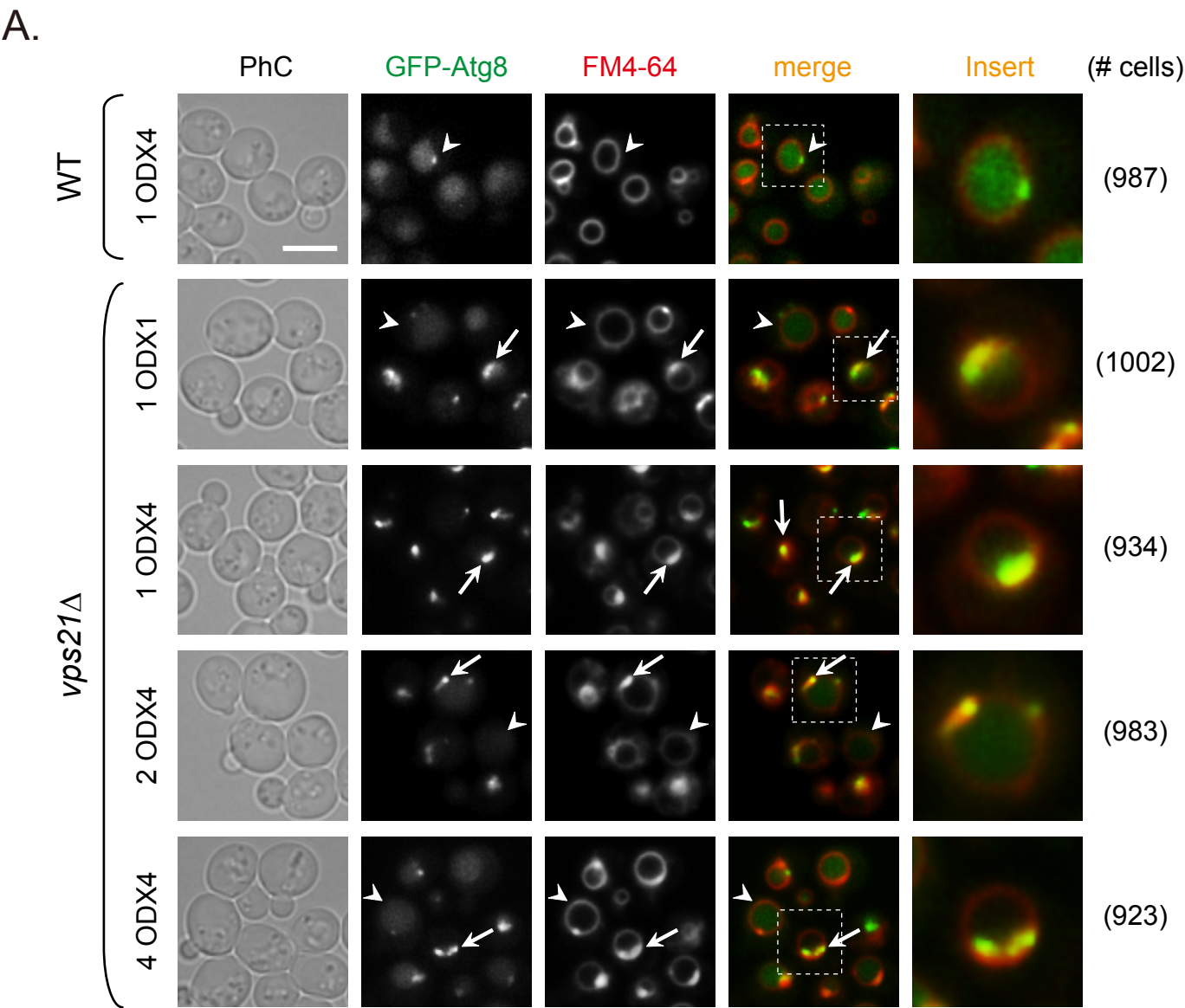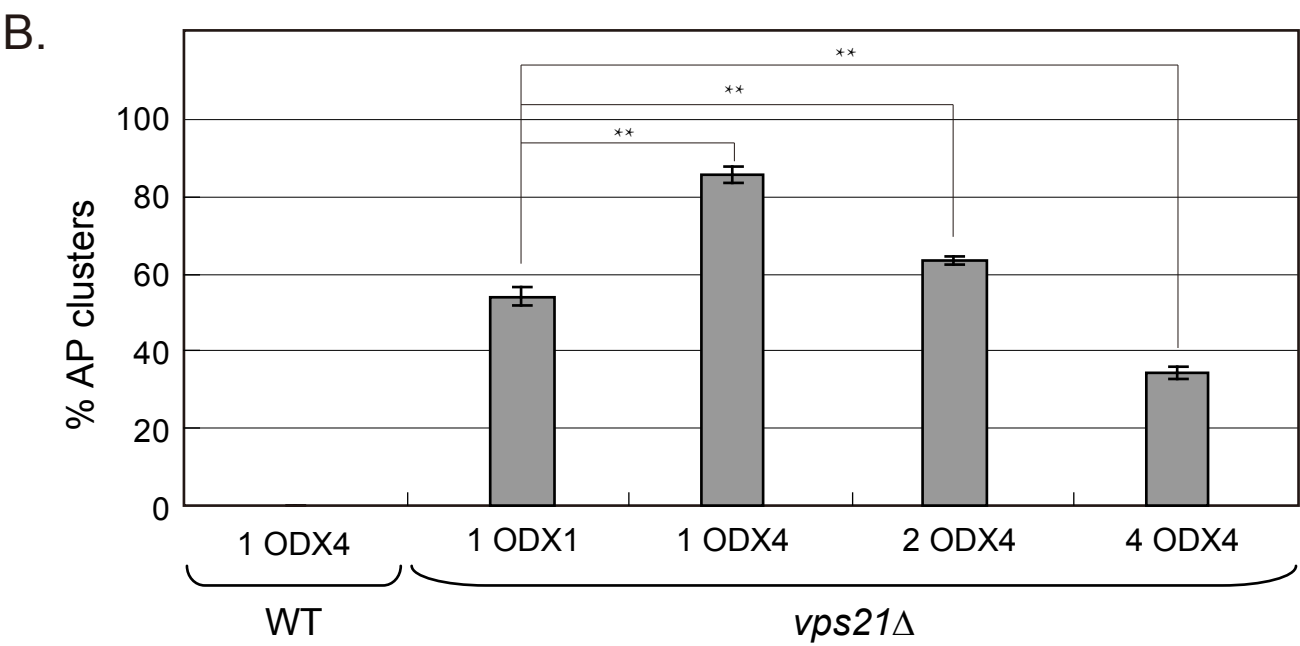

Supplement: S2 Fig — Wild type (WT) and vps21Δ cells expressing GFP-Atg8 were grown under different conditions for observing GFP-Atg8 and FM4-64 patterns. A. GFP-Atg8 and FM4-64 patterns in WT and vps21Δ cells. A single colony was inoculated into YPD and grown to the indicated OD600 for three successive re-inoculations as follows: from 0.03, 0.06, and 0.12 OD600 to 1, 2 and 4 of OD600, respectively (marked: 1 ODX4; 2 ODX4; 4 ODX4, respectively). Additionally, a single colony of vps21Δ cells was inoculated into YPD to reach 1 OD600 without successive re-inoculations (marked: 1 ODX1). All cultures were then inoculated in YPD at 0.06 OD600 and grown for 6 hours (with rotation at 200 rpm) to reach mid-log phase, washed with water, and shifted to SD-N medium for 2 hours. FM4-64 was added during the second hour before collecting the cells. The co-localization of Atg8 and FM4-64 was determined using live-cell fluorescence microscopy. Shown from left-to-right: strains, culture conditions, PhC, GFP, FM4-64, merge, insert, and the number of cells quantified for each strain (from 3 different experiments). Arrows indicate co-localizing clusters, arrowheads point to GFP-Atg8 localizing in FM4-64 stained vacuoles; bar, 5 μm. B. Quantification of cells with GFP-Atg8 clusters (%) from panel A in the two strains with indicated growth conditions (bottom). A higher percent of vps21Δ mutant cells that contain Atg8 clusters is observed when cells were grown to a lower OD600 (from ~35 to 85%), and when the cells were re-inoculated three times versus once (~55 to 85%). Columns represent mean, error bars represent STD, and P values, **, p <0.01. Results in this figure represent three independent experiments and are relevant to Fig 1. (PDF) [file pgen.1007020.s003.pdf]

Figure S3

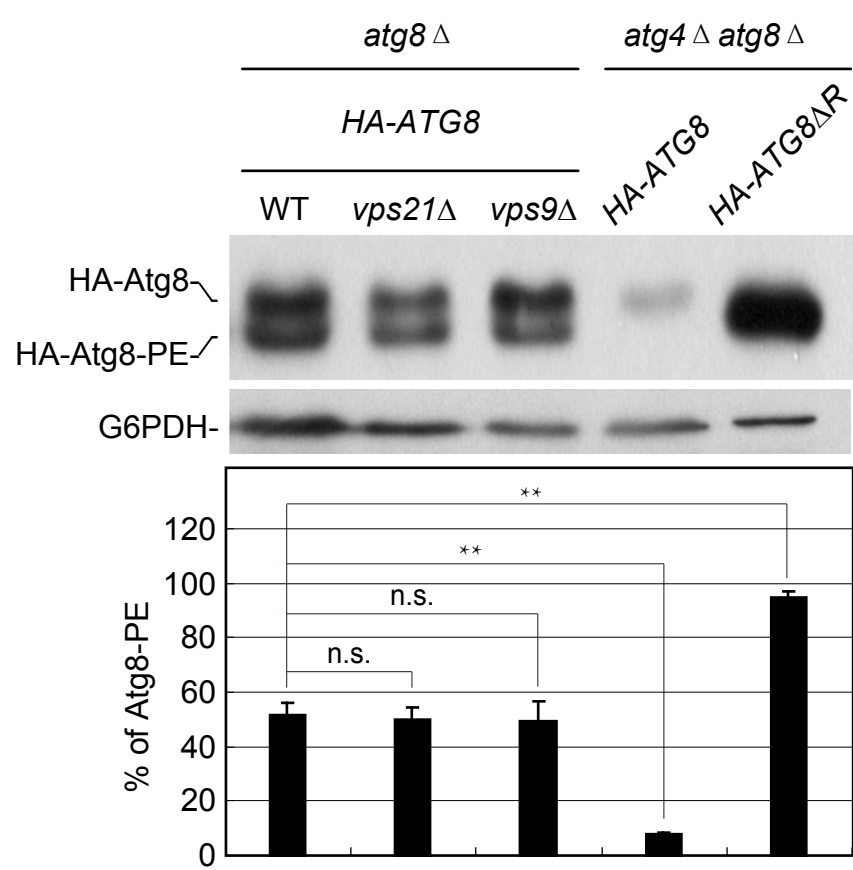

Supplement: S3 Fig — Cells deleted for ATG8 and expressing HA-Atg8 (or HA-Atg8ΔR) from the URA3 locus were grown and treated as in Fig 2. Immunoblot analysis was done using anti-HA antibodies. Shown from left to right: WT atg8Δ, vps21Δ atg8Δ, vps9Δ atg8Δ, all expressing HA-Atg8, and atg4Δ atg8Δ expressing HA-Atg8 or HA-Atg8ΔR. Shown from top to bottom: strain genotype, HA blot, G6PDH as a loading control and a bar graph showing the quantification of the Atg8-PE band as a percent of the total Atg8 protein. The level of Atg8-PE is similar in WT, vps21Δ and vps9Δ; atg4Δ serves as a negative control (with Atg8ΔR it can be lipidated even in the absence of Atg4). Bands were quantified for density and calculated as% of Atg8-PE accounted for total Atg8. P values, n.s., not significant; **, p<0.01. Experiments were repeated three times and representative blots are shown. (PDF) [file pgen.1007020.s004.pdf]

Figure S4

A.

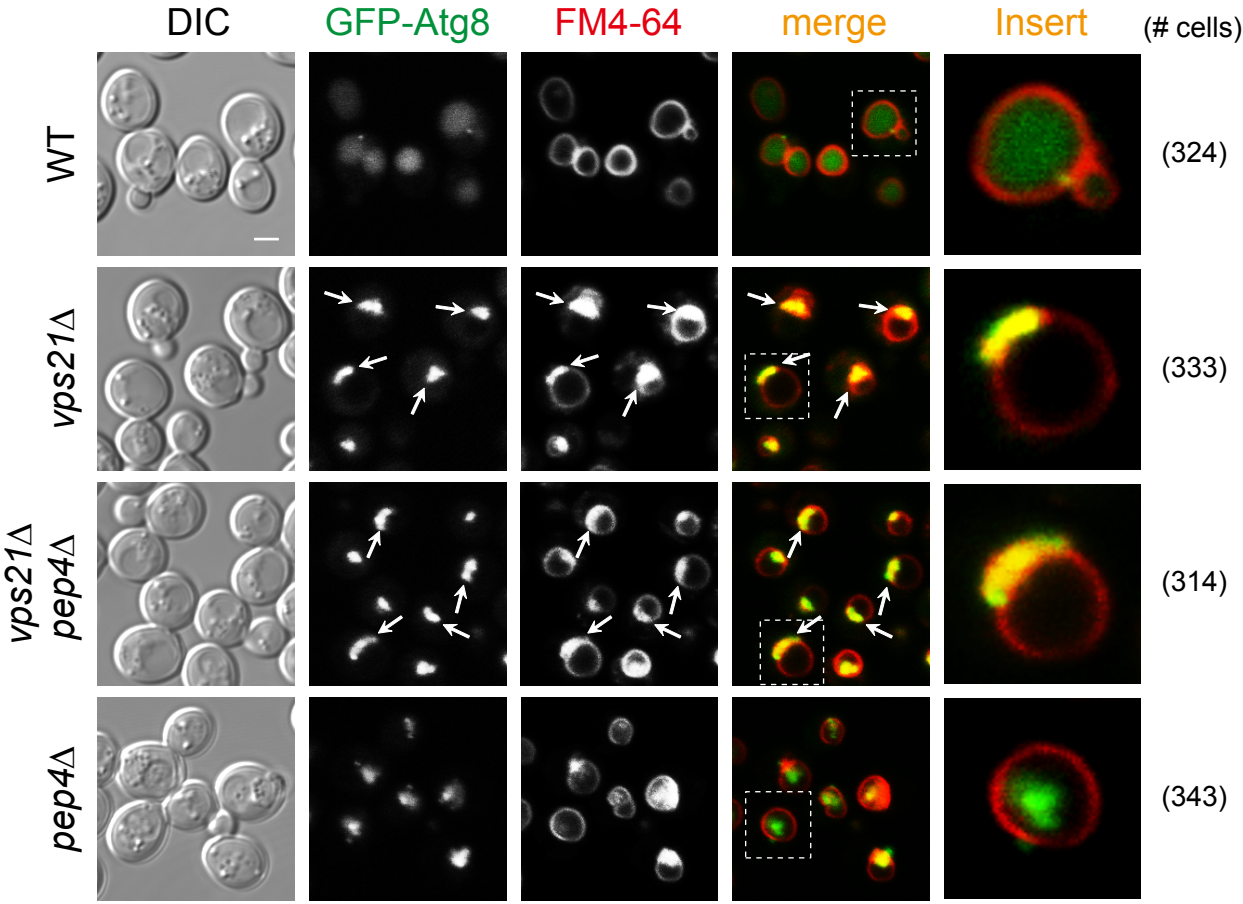

B.

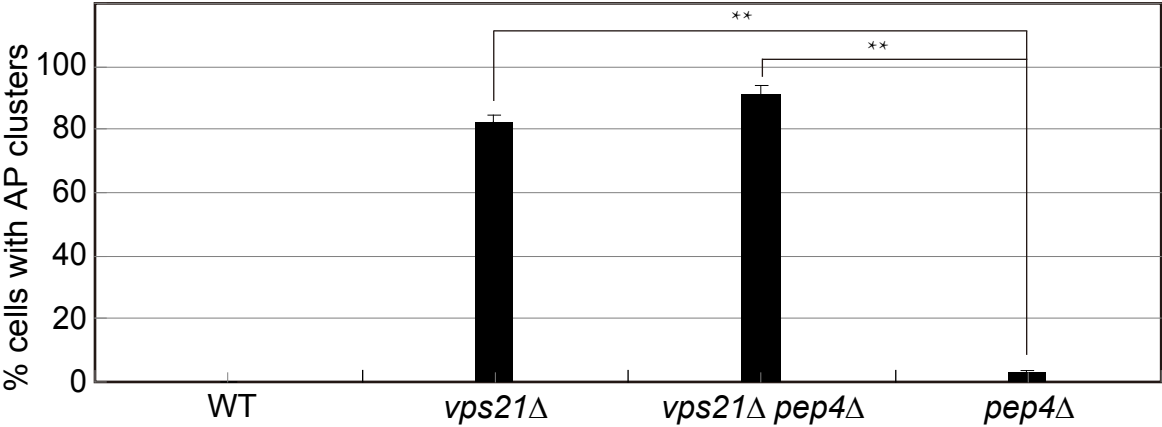

C.

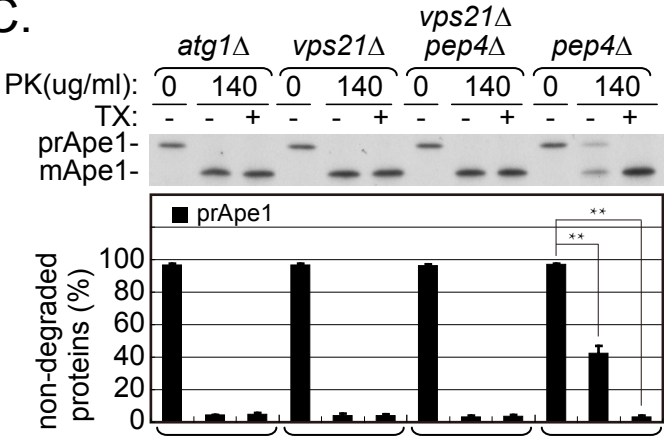

D.

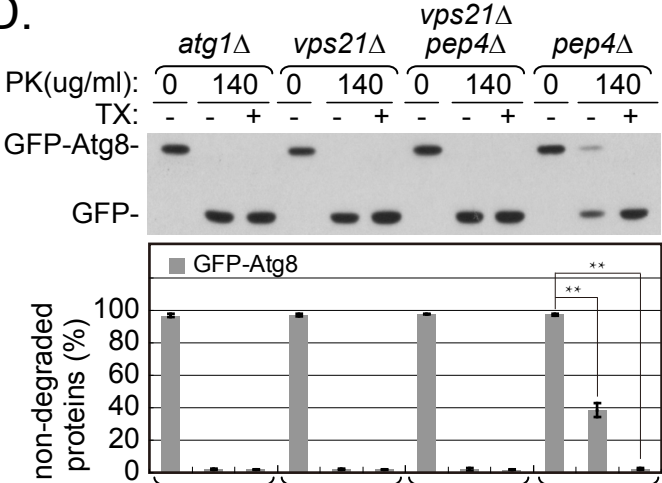

Supplement: S4 Fig — A. GFP-Atg8-labled APs accumulate in clusters near the vacuoles of vps21Δ and vps21Δ pep4Δ, but inside the vacuoles in pep4Δ, mutant cells. Yeast cells were grown, starved and visualized as in Fig 5A. Shown from left to right: strains, DIC, GFP, FM4-64, merge, insert, and number of cells visualized with GFP-Atg8. Shown from top to bottom: WT, vps21Δ, vps21Δ pep4Δ and pep4Δ. Arrows indicate co-localizing Atg8 and FM4-64 clusters; bar, 2 μm. 80–90% of the vps21Δ and vps21Δ pep4Δ mutant cells contain Atg8 clusters (see quantification in B). B. Quantification of results from A is shown as the percent of cells with GFP-Atg8 clusters in the indicated strains. Columns represent the mean, and error bars represent STD. C-D. Whereas sealed APs accumulate in pep4Δ, unsealed APs accumulate in vps21Δ and vps21Δ pep4Δ mutant cells. Protease protection analysis was done (as described in Fig 2) in the following strains (from left to right): atg1Δ (unprotected control), vps21Δ, vps21Δ pep4Δ, and pep4Δ. Shown from top to bottom: strain, -/+ PK, -/+ TX, Ape1 blot (C) or GFP blot (D), and quantification of non-degraded proteins (prApe1, black columns in C; GFP-Atg8, gray columns in D). Both prApe1 and GFP-Atg8 are protected from degradation in AP fractions isolated from pep4Δ, but not from vps21Δ and vps21Δ pep4Δ mutant cells. P values, **, p <0.01. Results in this figure represent three independent experiments. (PDF) [file pgen.1007020.s005.pdf]

# Figure S5

A.

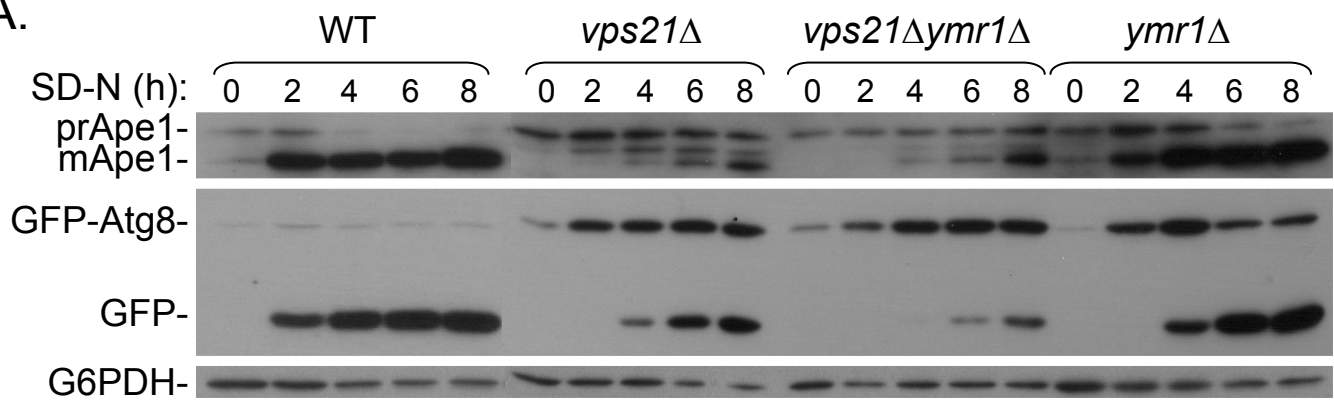

B.

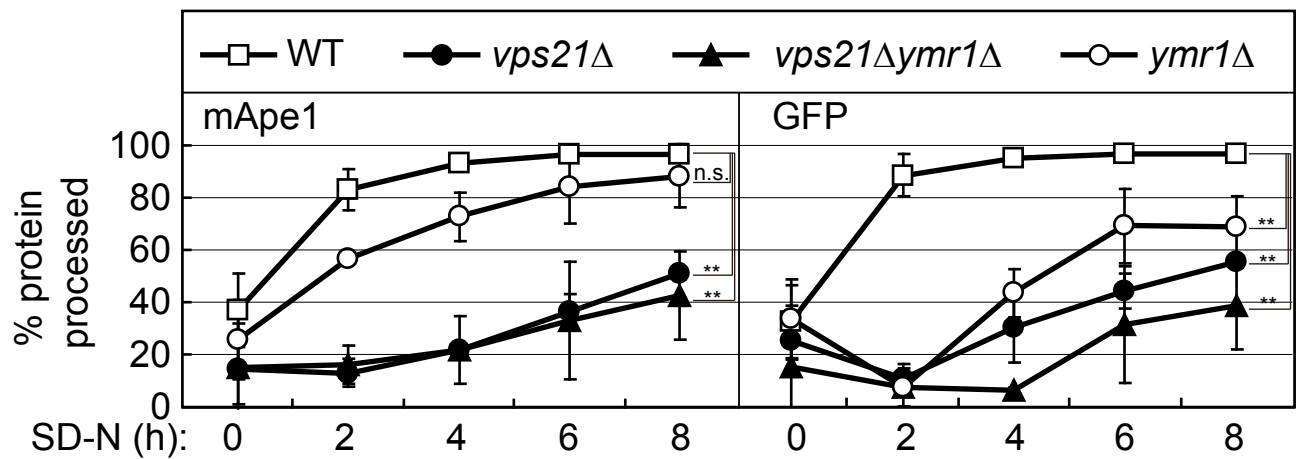

Supplement: S5 Fig — A. Cells expressing GFP-Atg8 from the chromosome were grown in YPD and shifted to SD-N for the indicated time. Processing of prApe1 to mApe1 and GFP-Atg8 to GFP was determined in lysates using immunoblot analysis with anti-GFP and anti-Ape1 antibodies, respectively. Shown from top to bottom: strain, time of starvation (0 to 8 hours), Ape1 blot, GFP blot, and G6PDH blot (loading control). B. Quantification of the percent of processed proteins from A is shown for mApe1 from prApe1 (left), and GFP from GFP-Atg8 (right), in WT (open squares), vps21Δ (closed circles), vps21Δ ymr1Δ (closed triangles), and ymr1Δ (open circles), mutant cells. Points on the graphs represent mean, error bars represent STD and P values for SD-N for 8 hours: n.s., not significant; **, p <0.01. Results in this figure represent three independent experiments and complement Fig 5. (PDF) [file pgen.1007020.s006.pdf]

# Figure S6

A.

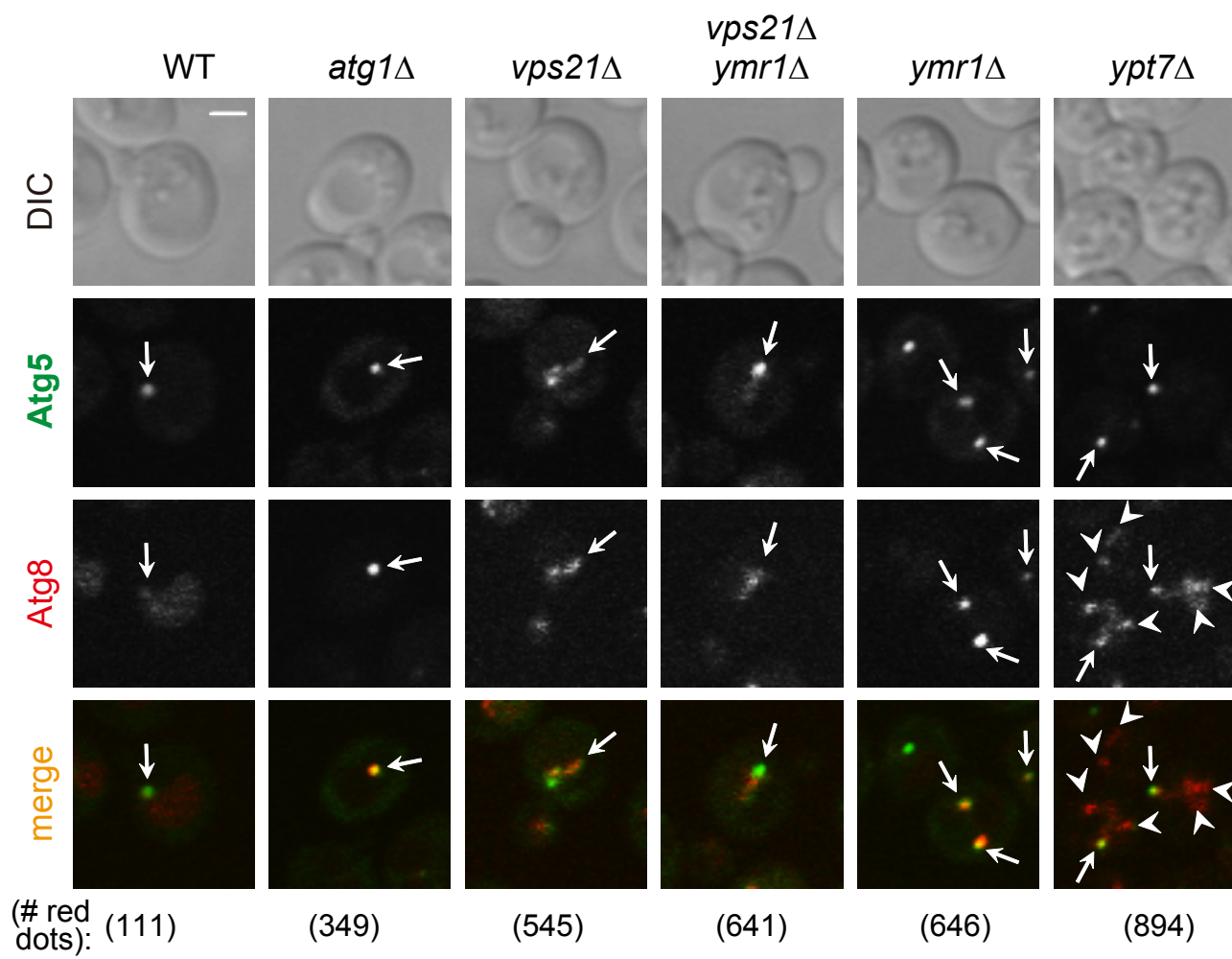

B.

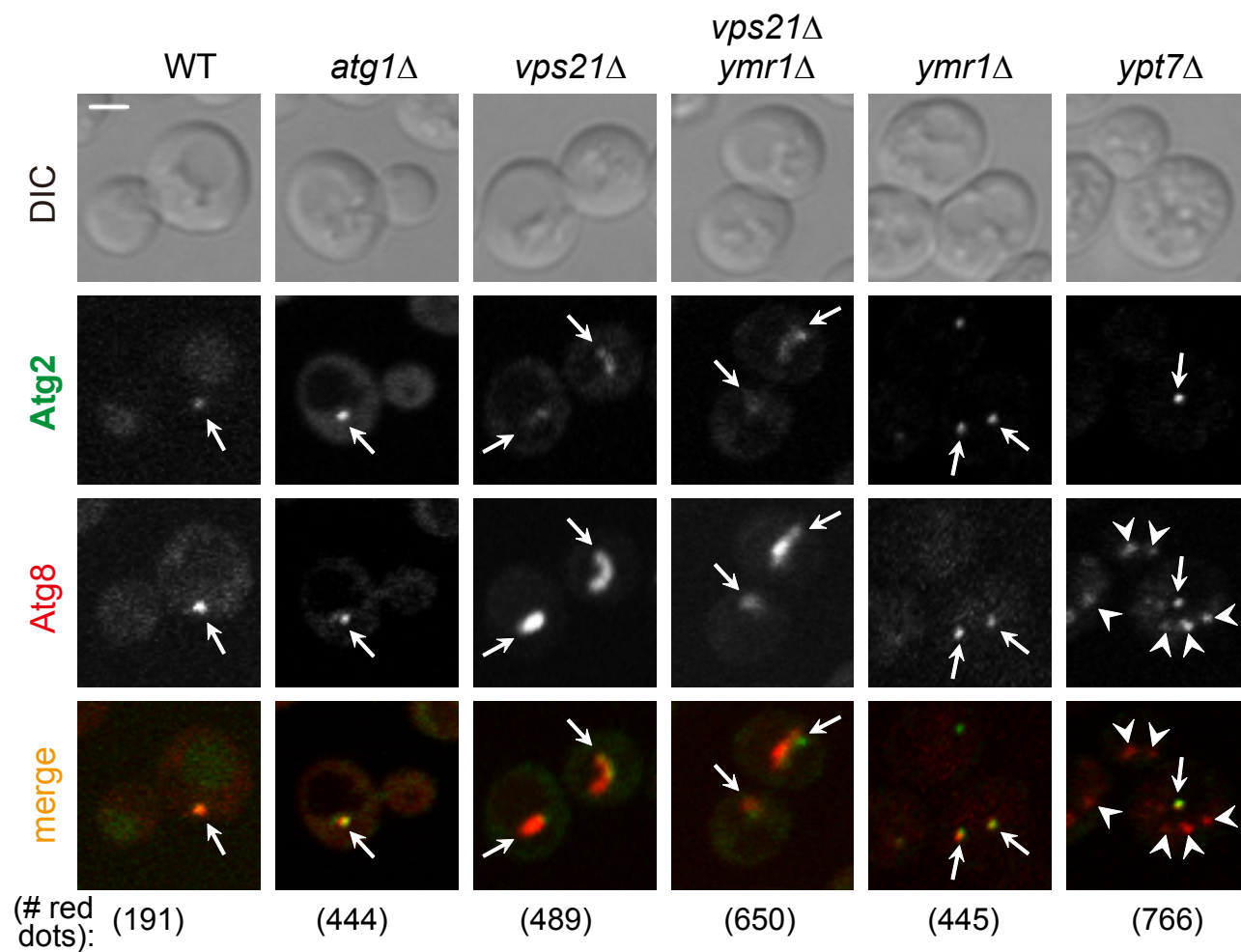

Supplement: S6 Fig — The co-localization of Atg5 (A), and Atg2 (B), with the AP marker mCherry-Atg8 was determined and presented as described in Figs 3 and 4; bar, 2 μm. Results in this figure represent three independent experiments and their quantification of results from this figure is shown in Fig 5F. (PDF) [file pgen.1007020.s007.pdf]

# Figure S7

A.

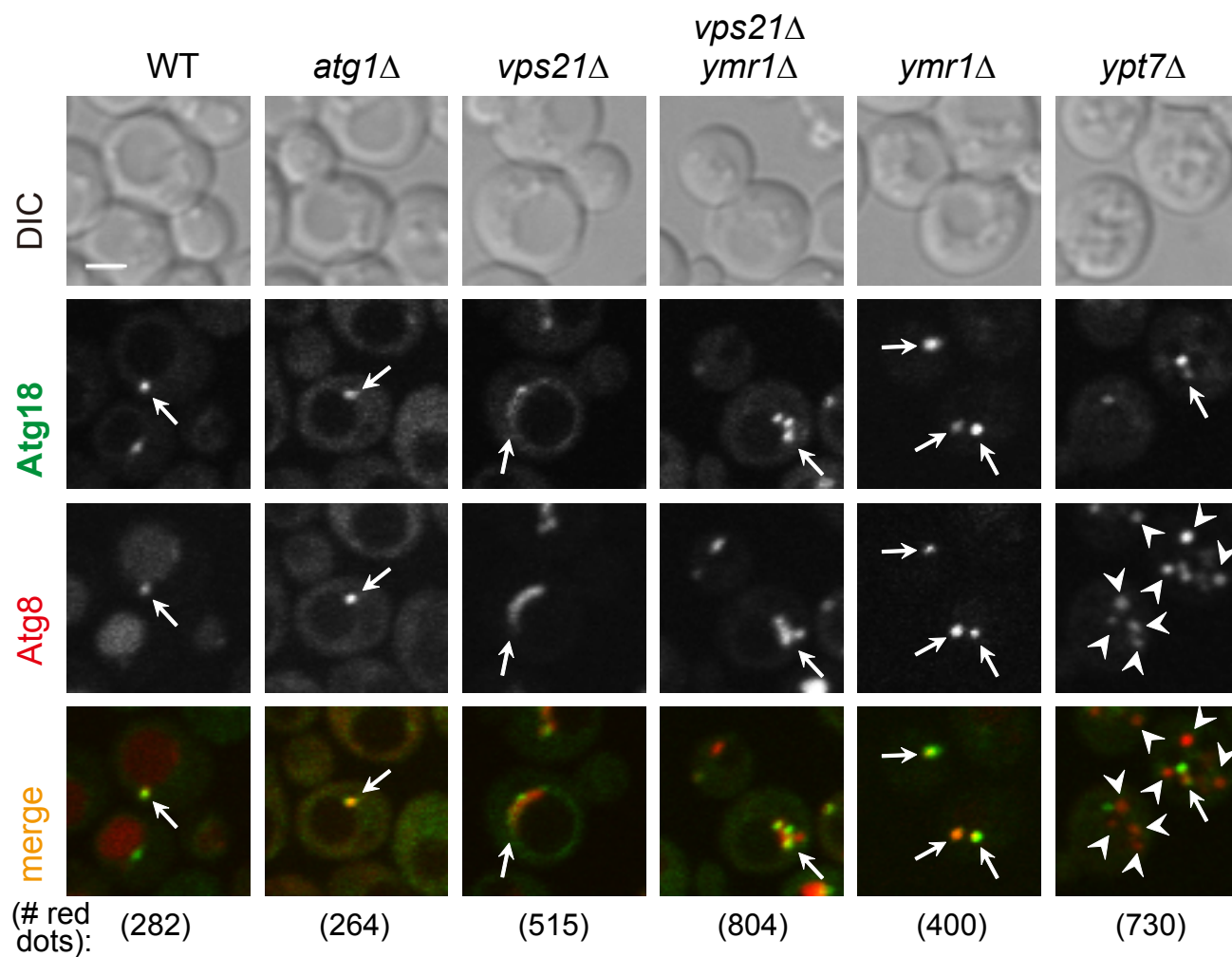

B.

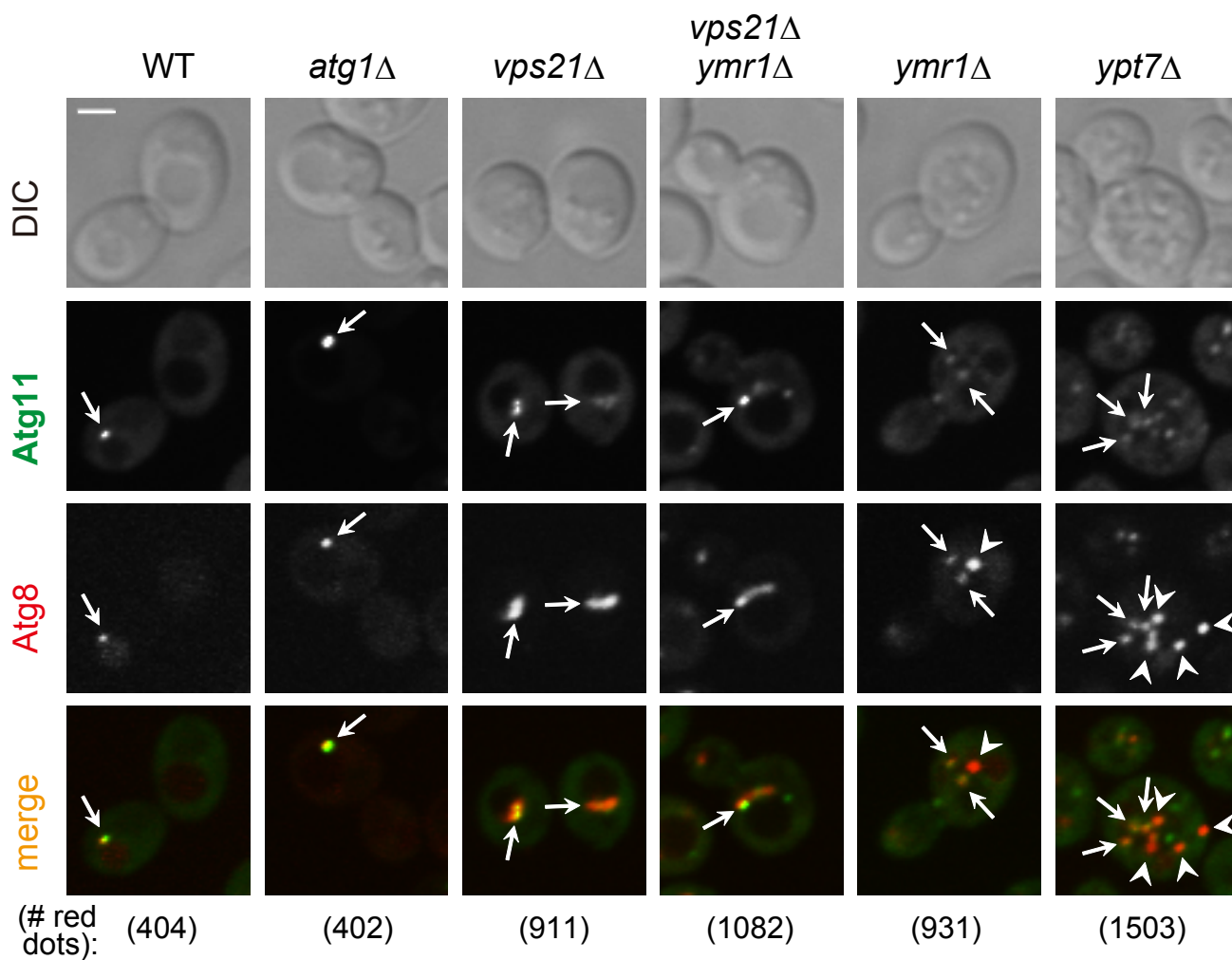

Supplement: S7 Fig — The co-localization of Atg18 (A), and Atg11 (B), with the AP marker mCherry-Atg8 was determined and presented as described in Figs 3 and 4; bar, 2 μm. Results in this figure represent three independent experiments and their quantification of results from this figure is shown in Fig 5F. (PDF) [file pgen.1007020.s008.pdf]
